# Nonameric Peptide Orchestrates Signal Transduction in the Activating HLA-E/NKG2C/CD94 Immune Complex as Revealed by All-Atom Simulations

**DOI:** 10.3390/ijms22136670

**Published:** 2021-06-22

**Authors:** Eva Prašnikar, Andrej Perdih, Jure Borišek

**Affiliations:** 1 National Institute of Chemistry, Hajdrihova 19, 1000 Ljubljana, Slovenia; eva.prasnikar@ki.si; 2 Graduate School of Biomedicine, Faculty of Medicine, University of Ljubljana, Vrazov Trg 2, 1000 Ljubljana, Slovenia; 3 Faculty of Pharmacy, University of Ljubljana, Aškerčeva 7, 1000 Ljubljana, Slovenia

**Keywords:** immune complex, molecular dynamics, immunology, NK cell, signal transduction

## Abstract

The innate immune system’s natural killer (NK) cells exert their cytolytic function against a variety of pathological challenges, including tumors and virally infected cells. Their activation depends on net signaling mediated via inhibitory and activating receptors that interact with specific ligands displayed on the surfaces of target cells. The CD94/NKG2C heterodimer is one of the NK activating receptors and performs its function by interacting with the trimeric ligand comprised of the HLA-E/β2m/nonameric peptide complex. Here, simulations of the all-atom multi-microsecond molecular dynamics in five immune complexes provide atomistic insights into the receptor–ligand molecular recognition, as well as the molecular events that facilitate the NK cell activation. We identify NKG2C, the HLA-E_α2_ domain, and the nonameric peptide as the key elements involved in the molecular machinery of signal transduction via an intertwined hydrogen bond network. Overall, the study addresses the complex intricacies that are necessary to understand the mechanisms of the innate immune system.

## 1. Introduction

Natural killer (NK) cells are a lymphocyte lineage with cytotoxic and cytokine-producing functions that are part of the innate immune system and defend the organism against various types of tumors and microbial infections [1,2,3]. They enable maternal adaptation to pregnancy [4] and regulate both innate and adaptive immune responses through reciprocal interactions with dendritic cells, macrophages, T cells, and endothelial cells [1,2]. NK cells are regulated by the activating (e.g., NKG2D/NKG2D, KIR2DS, CD94/NKG2C, -E, -H) and inhibitory (e.g., CD94/NKG2A, -B, KIR2DL, KIR3DL) surface receptors that recognize normally present, overexpressed or de novo expressed ligands on the surface of target cells [5,6,7]. The balance between the inhibitory and activating receptor signaling is one of the fundamental mechanisms of the immune system, as the predominant signal regulates the NK cell cytolytic function and ultimately influences the survival of the target cell [6].

NK cell receptors recognize the MHC class I molecules and belong to different receptor families. The latter include the killer cell Ig-like receptors, immunoglobulin-like transcripts, and the C-type lectin family, which consists of a heterodimer of CD94 and a representative of the NKG2x family of molecules (NKG2-A/B, -C, -E, -H, -F), except for the NKG2D protein, which exists as a homodimer. The ligands for the first two families include the classical (class Ia) HLA class I molecules and the nonclassical (class Ib) HLA-G protein, while the ligand for most of the CD94/NKG2x heterodimers is a nonclassical (class Ib) glycoprotein HLA-E [6]. The stable surface expression of the latter requires the formation of a heterotrimer comprised of a HLA-E heavy chain, its light beta-2-microglobulin (β2m) chain, and a specific nonameric peptide (hereafter referred to as a peptide) often derived from the signal sequences of other HLA class I molecules (e.g., HLA-A, -B, -C, -G) [6]. The NKG2x/CD94 receptor expression is not stable and can be regulated by cytokines [4].

The NKG2x/CD94 receptors can mediate the inhibitory or activating signals to the NK cells despite their high similarity. For example, the inhibitory NKG2A and activating NKG2C receptor proteins possess a 75% sequence identity, which is even higher for the extracellular domains responsible for the ligand recognition. The NKG2A protein performs its function via an immunoreceptor tyrosine-based inhibitory motif (ITIM) at its cytoplasmic tail [7,8]. On the other hand, the NKG2C protein lacks this signaling part and instead interacts with the associated adaptor protein DNAX activation protein of 12kDa (DAP12), which carries an immunoreceptor tyrosine-based activation motif (ITAM) responsible for the sequential NK cell activation [7,8]. The differences between the receptors also extend to their susceptibility to bind to HLA-E ligands. The affinity of HLA-E towards the activating NKG2C/CD94 receptor was found to be about 6-fold lower than the affinity for the inhibitory NKG2A/CD94 receptor, which was mainly attributed to the difference in residues: 165–168 (NKG2C) and 167–170 (NKG2A) [9].

Binding between the NKG2x/CD94 receptor and the HLA-E/β2m/peptide ligand strongly depends on the identity of the peptide incorporated into the ligand counterpart. Peptide G, derived from the signal sequence of the HLA-G molecule, most strongly regulates the signaling of NKG2C receptor and enables the activation of the NK cells. Such an outcome can be also achieved by the HLA-B27 leader peptide, although to a much lesser extent [10]. Furthermore, some reports have even indicated that peptide G is currently the only known peptide capable of potent NK cell activation [11]. In contrast, the presence of Cw7 and B7 peptides in the HLA-E/β2m/peptide ligand complex does not lead to NK cell activation [10]. Overall, it seems that the inhibition of the NK cells via NKG2A/CD94 can be achieved with a broader range of known peptides than activation via the NKG2C/CD94 receptor [10,11]. The exact reasons for this observation are currently unknown.

In light of the currently ongoing COVID-19 pandemic, the NKG2C receptor and the HLA-E allele were also associated with the severity of clinical manifestations of COVID-19. In hospitalized patients, the deletion of the activating NKG2C receptor encoding gene (*KLRC2*) and, to a lesser extent, the overexpression of the HLA-E*0101 allele were observed [12]. Moreover, an increased expression of the inhibitory NKG2A receptor was found in patients with severe COVID-19 [12]. On a similar note, the deletion of the NKG2C receptor and the expansion of the NKG2A**^+^** NK cells were reported in psoriasis, with the deletion of the *NKG2C* gene marked as a risk factor for psoriasis susceptibility [13]. Interestingly, the overexpression of NKG2C has also been detected in high-functioning autism spectrum disorders and may have deleterious consequences for the central nervous system [14]. An altered NKG2C expression is also present in first-episode psychosis [15], and a higher percentage of the NKG2C**^+^** cells has been reported in patients with chronic obstructive pulmonary disease with frequent exacerbations and a reduced lean mass [16].

Mutagenesis studies [17,18] have clarified some of the molecular details of the receptor (NKG2C/CD94)–ligand (HLA-E/β2m/peptide) intermolecular interactions, and further binding and cytotoxicity assays [10,11] have uncovered the roles of specific peptides on the overall outcome of the NK cells. Unfortunately, these studies are quite scarce for the activating NKG2C/CD94/HLA-E/β2m/peptide immune complex, compared to the inhibitory one involving the NKG2A receptor protein. In addition, the crystal structure of this system has not been determined to date. Thus, the mechanistic details and subtleties of the successful signal transduction responsible for small peptide-mediated NK cell activations remain elusive and call for additional investigation.

In this work, we performed all-atom molecular dynamics (MD) simulations of HLA-E/β2m/NKG2C/CD94 immune complexes containing known nonameric peptides that either activate or inhibit NK cells, to shed some light on the mechanistic details that govern the molecular recognition of the complexes and the subsequent successful signal transduction at the atomistic level. The simulations helped us to evaluate the global dynamics of the complex multicomponent systems, elucidate the fundamental intermolecular interactions, and further substantiate the obtained findings with energy calculations.

## 2. Results

**Figure 1:**
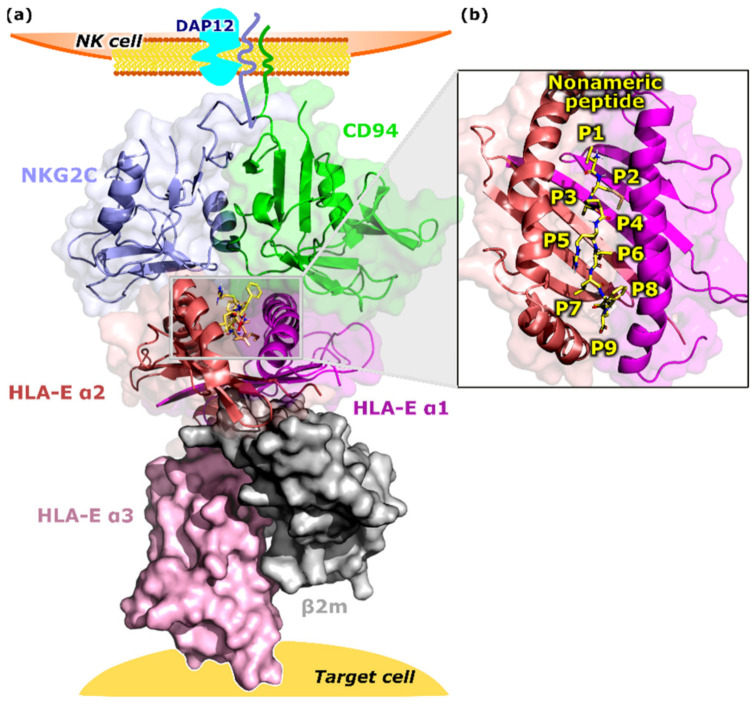
Overview of the HLA-E/peptide/β2m/NKG2C/CD94 immune complex assembled on the surfaces of NK and target cells. (**a**) The multicomponent complex was comprised of a HLA-E heavy chain divided into three alpha domains (α1–α3), HLA-E light chain β2m, nonameric peptide, CD94 and NKG2C proteins (magenta, salmon, and pink, grey, yellow, green, and blue, respectively). CD94 and NKG2C transmembrane regions and DAP12 protein are only schematically presented. (**b**) Magnified binding pocket of the nonameric peptide located between the HLA-E α1 and α2 domains. HLA-E—HLA class I histocompatibility antigen, alpha chain E, β2m—Beta-2-microglobulin, NKG2C—NKG2-C type II integral membrane protein, CD94—Natural killer cells antigen CD94, NK cell—Natural killer cell, DAP12—DNAX activation protein of 12kDa.

We studied the structural features and the dynamic behavior of five models of immune complexes based on the available crystal structures of the extracellular domains of the human CD94/NKG2A in a complex with HLA-E (PDB ID 3CDG). The models included all the resolved components consisting of CD94, HLA-E, β2m, and a bound peptide nested between the α1 and α2 domains of the HLA-E ligand, while the protein NKG2A was replaced by the generated homology model of the NKG2C protein (Figure 1 and Movie in Supplementary File S2). Both considered extracellular sequences of NKG2C (111–230) and NKG2A (113–232) proteins display a high level of identity, as they differ only in six residues. Thus, the replacement was rather straightforward.

The multicomponent HLA-E/peptide/β2m/NKG2C/CD94 models differed only in the sequence of the bound peptide with: (i) the peptide from the HLA-G leader sequence (**COM^+^_G_**), (ii) the peptide from the HLA-B27 (**COM^~^_B27_**), (iii) the HLA-B7 signal sequence peptide (**COM^−^_B7_**), (iv) the HLA-Cw7 signal peptide (**COM^−^_Cw7_**), and finally, (v) a hypothetical immune complex deprived of its peptide (**COM_apo_**). Notably, the **COM^+^_G_** model mediated the NK cell activation, whereas the currently available data for the **COM^~^_B27_** action are inconclusive [10,11]. According to the experiments, the immune complexes represented by the **COM^−^_B7_** and **COM^−^_Cw7_** models did not result in the NK cell activation. As a negative control and to further study the overall role of the peptide in the complex, we also constructed a **COM_apo_** model without the bound peptide (Table 1).

### 2.1. Peptide, NKG2C, and HLA-E_α2_ Serve as Key Complex Elements in the Signal Transduction

MD simulations that lasted for several microseconds with a total time of ~6 µs did not detect any major conformational changes in any of the five studied immune complexes. This observation could suggest that a “lock and key”-like engagement takes place between the HLA-E/peptide/β2m ligand and the NKG2C/CD94 receptor that has also previously been suggested for the NKG2A system [19].

**Figure 2:**
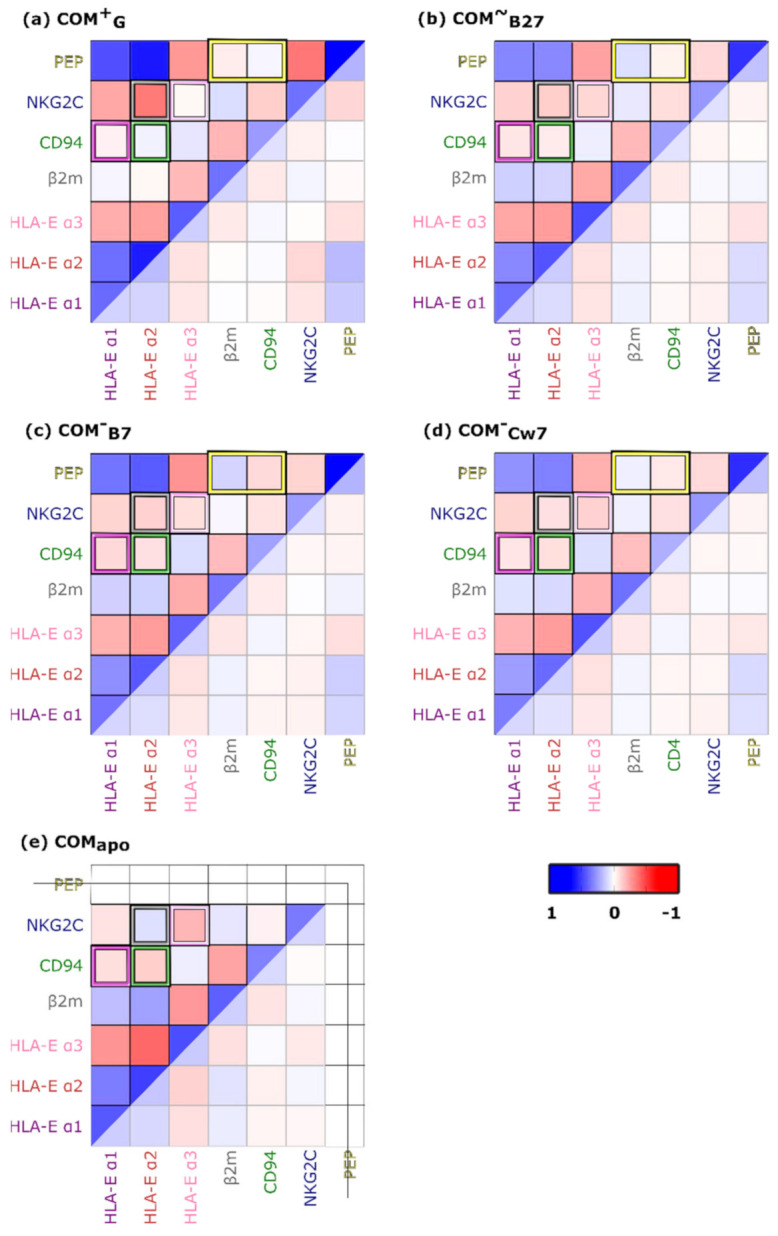
Simplified cross-correlation matrices for (**a**) **COM^+^_G_**, (**b**) **COM^~^_B27_**, (**c**) **COM^-^_B7_**, (**d**) **COM^-^_Cw7_**, and (**e**) **COM_apo_** simulated models of the immune complexes. In the yellow brackets the peptide (anti)correlations with proteins CD94 and β2m are highlighted, while the grey brackets feature the NKG2C–HLA-E_α2_, the pink brackets feature the NKG2C–HLA-E_α3_, the violet brackets feature the CD94–HLA-E_α1_, and the green brackets feature the CD94–HLA-E_α2_ relations. The per-residue Pearson’s coefficients (*CCijs*) cross-correlation matrix was derived from the mass-weighted covariance matrix calculated over the last 1 μs of the classical molecular dynamics trajectories. *CCijs* values range from −1 (red, anticorrelated motions) to +1 (blue, correlated motions) and are summed and normalized for each pair of proteins/domains considered to obtain density correlation scores (*CSs*). For the cross-correlation matrices calculation HLA-E α1, α2, and α3 domains, β2m, CD94, NKG2C and peptide are considered. HLA-E—HLA class I histocompatibility antigen, alpha chain E, β2m—Beta-2-microglobulin, NKG2C—NKG2-C type II integral membrane protein, CD94—Natural killer cells antigen CD94.

Subsequently, we investigated the internal dynamics in more detail and generated the cross-correlation matrices (*CCji*) for the system’s components, which were simplified for clearer representation. These revealed significant differences in the correlation patterns between the system´s receptor and ligand proteins. Namely, in the **COM^+^_G_** model, the HLA-E_α2_ domain was more anticorrelated with the NKG2C receptor protein, in addition to being more correlated with the CD94 receptor partner compared to all other investigated models. In **COM^~^_B27_**, **COM^−^_B7_**, and **COM^−^_Cw7_** models, both the CD94 and NKG2C parts of the receptor were shown to be anticorrelated with HLA-E_α2_. Finally, in the peptide-free **COM_apo_** model, the HLA-E_α2_ was correlated with NKG2C, and anticorrelated with CD94 (Figure 2 and Supplementary Figure S1). Hence, it appears that with the incorporation of the peptide into the system, the correlation between NKG2C and HLA-E_α2_ is lost. The generated *CCji* also revealed that during the ligand–receptor recognition and the activating signal transduction, which according to experimental data occurs in the **COM^+^_G_** model, the CD94 protein of the NKG2C/CD94 receptor becomes correlated with HLA-E_α2_, while the NKG2C–HLA-E_α2_ anticorrelation becomes even more pronounced than in other models. Furthermore, we observed that the CD94–HLA-E_α1_ and NKG2C–HLA-E_α3_ anticorrelations decreased in the **COM^+^_G_** model (Figure 2 and Supplementary Figure S1).

Cross-correlation matrices further uncovered that the nonameric peptide G in the **COM^+^_G_** model was slightly correlated with the CD94 protein and anticorrelated with the β2m part of the ligand, which was reversed in the other three peptide-containing models **COM^~^_B27_**, **COM^−^_B7_**, and **COM^−^_Cw7_** (Figure 2 and Supplementary Figure S1). This interesting difference in the peptide influence on the system could be explained by the more pronounced anticorrelated relationship of the β2m light chain and the more correlated behavior of the CD94 protein with the α1- and α2-domains of the HLA-E protein with which the activating peptide G is in contact. On the other hand, the CD94 protein was more correlated with the G peptide and slightly more anticorrelated with NKG2C in the **COM^+^_G_** model (Figure 2 and Supplementary Figure S1). Taken together, the observed conformational behavior in the analyzed trajectories implies that the bound G peptide induces a disturbance in the interactions between the NKG2C–CD94 receptor partners, which in turn could lead to altered communication between the NKG2C protein and the adaptor protein DAP12, which is ultimately responsible for the NK cell activation.

Overall, in the simulated **COM^+^_G_** model, more pronounced correlated and anticorrelated associations between its parts were observed. This was particularly noticeable for the NKG2C–HLA-E_α2_, NKG2C–peptide, and peptide–HLA-E_α2_ contacts (Figure 2 and Supplementary Figure S1). This suggests that peptide, NKG2C, and HLA-E_α2_ components of the system act as key elements in further signal transduction. In this respect it could be further assumed that the peptide influences the NKG2C receptor protein via its engagement with the ligand HLA-E_α2_ domain, suggesting a possible crosstalk between them.

### 2.2. HLA-E_α2_ Domain May Facilitate a Crosstalk between the NKG2C Protein and the Peptide

**Figure 3:**
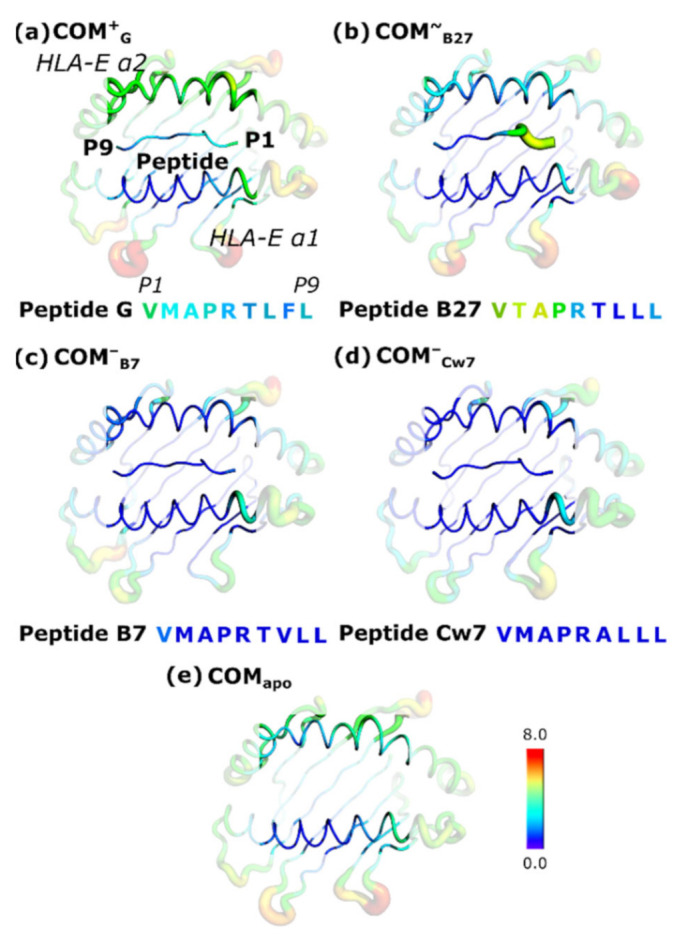
B-factor representation of the atomic Root Mean Square Fluctuations (RMSF) of the HLA-E α1 and α2 domains, and the bound peptide. (**a**) **COM^+^_G_**, (**b**) **COM^~^_B27_**, (**c**) **COM^-^_B7_**, (**d**) **COM^-^_Cw7_**, and (**e**) **COM_apo_**. The peptide sequences in one letter code are colored according to the corresponding B-factor. The parts of HLA-E_α1_ and HLA-E_α2_ domains that are not in the vicinity of the peptide and receptor proteins are shaded. HLA-E—HLA class I histocompatibility antigen, alpha chain E.

To further investigate the potential role of the HLA-E_α2_ domain in the NKG2C-peptide crosstalk, we analyzed the flexibility patterns of all the complexes. The RMSF analysis revealed that the alpha-helix of the HLA-E_α2_ domain located in the proximity of the peptide was more flexible in the **COM^+^_G_** model, while it was more rigid in the other peptide including models **COM^~^_B27_**, **COM^−^_B7_**, and **COM^−^_Cw7_**. In the **COM^+^_G_** immune complex, this alpha-helix of the HLA-E_α2_ domain was also more flexible in comparison to the alpha helix of the HLA-E_α1_ domain located in the proximity of the peptide. However, similar observations were made for the models **COM^~^_B27_** and **COM_apo_** (Figure 3).

The peptide was most flexible in the **COM^~^_B27_** model, though in the **COM^+^_G_** model a flexibility of the peptide G was also detected. Peptides included in the **COM^−^_B7_** and **COM^−^_Cw7_** models were predominantly rigid (Figure 3). These motility patterns were also confirmed by the peptide RMSD calculations (Supplementary Figure S2). When we compared the patterns of peptides B27 and G, we noticed that peptide B27 is mainly flexible at its N-terminal side, whereas peptide G also has a higher flexibility at its C-terminal side.

Since the studied models differ only in the peptide identity, this may suggest that the peptide could be responsible for the observed changes in the HLA-E_α2_ domain flexibility. The comparison of the **COM^+^_G_**, **COM^~^_B27_**, **COM^−^_B7_**, and **COM^−^_Cw7_** models with the **COM_apo_** model shows that the peptides derived from the leader sequences of HLA-Cw7, HLA-B7, and HLA-B27 rigidify the HLA-E_α2_ domain, while the peptide G, derived from HLA-G leader sequence increases its flexibility (Figure 3). This observation further substantiated the assumption that the peptide influences the increased flexibility of the HLA-E_α2_ domain which might subsequently facilitate the crosstalk with the NKG2C part of the NKG2C/CD94 receptor to active the NK cell.

### 2.3. Particular Hydrogen Bonds Could Be Involved in Activating Signal Transduction

In addition to the abovementioned “lock and key”-like binding behavior observed between the NKG2C/CD94 receptor and the HLA-E/β2m/peptide ligand, the similarities between the NKG2C and NKG2A-peptides containing the immune complexes extend to the observed hydrogen bond networks. Analyzing the most representative clusters obtained from the MD simulation trajectories and the occurrence of H-bonds, we noticed several H-bond interactions that have been previously reported for the HLA-E/β2m/peptide/NKG2A/CD94 crystal structure [19], namely, the P5–Ser110^CD94^, P5–Glu152^HLA-E^, and P6–Gln112^CD94^ interactions (Table 2 and Supplementary Table S1) [19].

**Figure 4:**
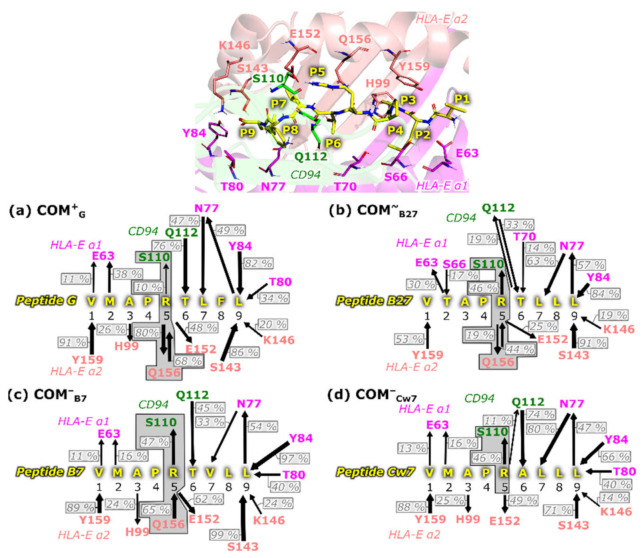
Schematic summary of the bound peptide interacting residues with HLA-E_α1_, HLA-E_α2_ and CD94 proteins for the models (**a**) **COM^+^_G_**, (**b**) **COM^~^_B27_**, (**c**) **COM^−^_B7_**, and (**d**) **COM^−^_Cw7_**. Only hydrogen (H) bonds with occurrences higher than 10% are depicted. The arrows point in the direction of residues acting as H-bond acceptors. The arrow line thickness represents the H-bond frequencies (stated in the brackets), where the thickest line represents the highest frequency. HLA-E—HLA class I histocompatibility antigen, alpha chain E.

**Figure 5:**
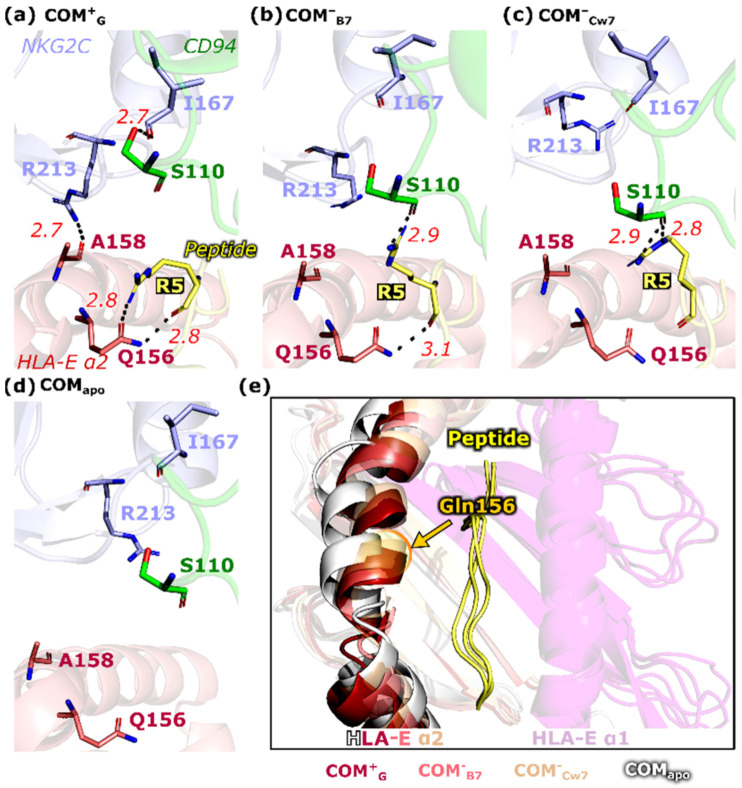
Representation of the key H-bond interaction differences observed between the simulated models. The hydrogen bonds between the putative key residues S110, Q156, A158 and the peptide R5 residue are involved in the signal transduction for models (**a**) **COM^+^_G_**, (**b**) **COM^−^_B7_**, (**c**) **COM^−^_Cw7_**, and (**d**) **COM_apo_**. (**e**) The alignment of the HLA-E_α2_ of the simulated models with a conclusive effect on the NK cells (**COM^+^_G_**, **COM^−^_B7_**, and **COM^−^_Cw7_**) and COM_apo_ with the highlighted position of the Gln156^HLA-E^ residue. Data is derived from the most representative cluster of the last 500 ns of production MD run. HLA-E—HLA class I histocompatibility antigen, alpha chain E, NKG2C—NKG2-C type II integral membrane protein, CD94—Natural killer cells antigen CD94.

In general, a similar H-bonding network of peptides within the system is present in all models (Figure 4) allowing no distinction between the **COM^+^_G_** model that mediated the NK cell activation signal and the models in which the activation was ambiguous (**COM^~^_B27_**,) or absent (**COM^−^_B7_**, and **COM^−^_Cw7_**). However, arginine at position five (P5) forms H-bonds with the HLA-E_α2_ domain and the CD94 protein with different frequencies. Namely, the occurrence of the P5–Gln156^HLA-E^ H-bond is most frequently present in model **COM^+^**_G_, whereas the P5–Ser110^CD94^ H-bond is the least present (Figure 4). Furthermore, in the **COM^+^_G_** immune complex, the P5 basic arginine side chain is oriented toward the HLA-E_α2_ domain, whereas in the other models it is extended toward the CD94 protein (Figure 5 and Figure S3).

Since the Ser110^CD94^ residue is strategically positioned at the NKG2C–CD94 receptor interface, its interaction with the peptide might lead to changes in the intermolecular relations between the NKG2C/CD94 receptor proteins resulting in the aforementioned higher anticorrelation between them. Indeed, the Ile167^NKG2C^–Ser110^CD94^ H-bond was found to be present in 34% of the last 1 μs of the production run of the **COM^+^_G_** immune complex and only in 9%, 6%, and 2% for the **COM^~^_B27_**, **COM^−^_B7_**, and **COM^−^_Cw7_** models, respectively, whereas in the **COM_apo_** model the H-bond was present in less than 0.1% of the trajectory. The formation of this H-bond was confirmed in the most representative cluster of the **COM^+^_G_** model derived from the last 500 ns of the simulation (Figure 5), as well as in an independent distance measurement where the H-bond was present especially in the last half of the production run (Supplementary Figure S4).

Furthermore, we noticed that Glu156^HLA-E^ and P5 residues form more H-bonds in **COM^+^_G_** compared to other models, which could influence the positioning and flexibility of the HLA-E_α2_ domain. Indeed, the alignment of the most representative cluster revealed a slight deviation in the (in)outward positioning of the HLA-E_α2_ α-helix in relation to the peptide and the HLA-E_α1_ domain, especially in the close surroundings of the Glu156^HLA-E^ residue (Figure 5e and Supplementary Figure S3). The H-bond analysis identified another contact at the receptor–ligand interface, namely the Arg213^NKG2C^-Ala158^HLA-E^ interaction, which was more present in the **COM^+^_G_** model compared to the other simulated systems (Supplementary Figure S5). Taken together, the H-bond analysis also indicates that the peptide could influence the interactions occurring between the NKG2C protein and HLA-E_α2_, which implies a crosstalk between the peptide and the NKG2C protein via the HLA-E_α2_ domain.

### 2.4. Energy Calculations Expose Favorable Hotspots

Binding free energies (ΔG_b_) were calculated between the peptide and the rest of the complex (HLA-E/β2m/NKG2C/CD94), and between the receptor (NKG2C/CD94) and the ligand (HLA-E/β2m/peptide) pairs, applying the molecular mechanics-generalized born surface area (MM-GBSA) method [20] and performing both a pairwise and per-residue decomposition of ΔG_b_. To verify the results, we also recalculated the energies using the gmx energy module of the Gromacs2016 [21] software package.

The pairwise decomposition of the binding free energies (ΔG_b_) calculated between the peptide and the rest of the complex showed that the peptide residues P4, P5, P6, and P8 form the most important energetic interactions with the NKG2C/CD94 receptor. This decomposition also revealed that the peptide residues P1, P5, and P9 form the strongest interactions with the HLA-E/β2m ligand (Supplementary Table S2). Excitingly, among the detected key peptide–receptor and peptide–HLA-E interactions are several that were previously reported for the NKG2A variant of the immune complex (see Supplementary Table S2, marked in bold) [19].

The aforementioned H-bond interactions between the peptide arginine P5 and the Ser110^CD94^ and Gln156^HLA-E^ residues were additionally verified on the energetic level by this pairwise decomposition of binding free energy. Indeed, the P5–Gln156^HLA-E^ interaction is energetically the most important interaction in the **COM^+^_G_** system while its importance decreases in the **COM^~^_B27_**, **COM^−^_B7_**, and **COM^−^_Cw7_** models (Supplementary Table S2). In contrast, the P5–Ser110^CD94^ H-bond seems to be the least important energetically in the **COM^+^_G_** model compared to the other three peptide-containing models. (Supplementary Table S2).

The subsequent per-residue decomposition of ΔG_b_ between the peptide and the rest of the system revealed that the peptide residues P2, P8, and P9 generally represent the strongest contributors to ΔG_b_, whereas the P1, P4, and P3 residues contribute the least (Supplementary Table S3). In previous studies of the analogous inhibitory HLA-E/β2m/peptide/NKG2A/CD94 immune complex, P2 and P9 peptide residues were proclaimed as the primary anchor positions [22], while the P8 residue, together with the P5 residues, putatively forms the majority of contacts with the receptor part of the complex [23]. Furthermore, the **COM^+^_G_** model displays the most favorable ΔG_b_ for the peptide–complex binding, compared to the other peptide-including models **COM^~^_B27_**_,_ **COM^−^_B7_**, and **COM^−^_Cw7_**, which showed similar binding free energies (Supplementary Table S4).

**Figure 6:**
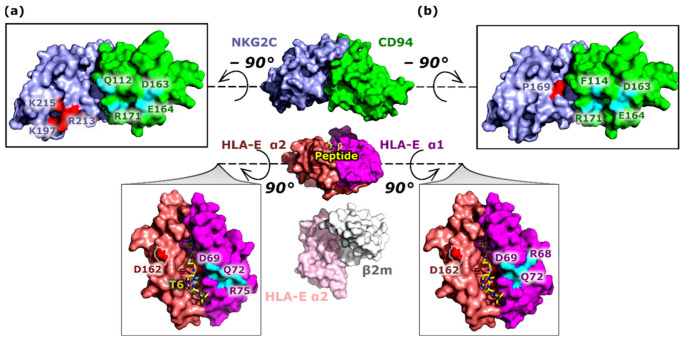
Key contacts underlying the receptor–ligand interface interactions. Residues with the greatest contribution to the binding free energy between the ligand-HLA-E/β2m/peptide (shown in magenta, salmon, pink, gray and yellow, respectively) and receptor-NKG2A/CD94 (in blue and green, respectively) with (**a**) pairwise and (**b**) per-residue decomposition, shown in surface and licorice representations (for the peptide). Residues are colored in red and cyan for the HLA-E_α2_–NKG2C and HLA-E_α1_–CD94 interacting protein pairs, respectively. The number of the colored residues on the receptor surface indicates the importance of the CD94 protein in the receptor–ligand interactions. HLA-E—HLA class I histocompatibility antigen, alpha chain E, β2m—Beta-2-microglobulin, NKG2C—NKG2-C type II integral membrane protein, CD94—Natural killer cells antigen CD94.

The plausible key interactions in the NKG2C/CD94/HLA-E/β2m/peptide complex were identified by performing a pairwise decomposition of ΔG_b_ calculated between the NKG2C/CD94 receptor and the HLA-E/β2m/peptide ligand pairs. All four peptide including models **COM^+^_G_**, **COM^~^_B27_**_,_ **COM^−^_B7_**, and **COM^−^_Cw7_** shared seven interactions, namely Asp69^HLA-E^-Arg171^CD94^, Arg75^HLA-E^-Asp163^CD94^, Asp162^HLA-E^-Arg213^CD94^, Gln72^HLA-E^-Glu164^CD94^, Asp162^HLA-E^-Lys215^NKG2C^, Asp162^HLA-E^-Lys197^NKG2C^, and Thr6^P6^-Gln112^CD94^ (Table S5 and Figure 6). On the other hand, the per-residue decomposition showed either ligand or receptor residues with the highest contribution to the ΔG_b_, obtained by summing its interactions over all the residues in the system. These residues can also be referred to as hotspots [24]. Again, all four peptide including models shared nine hotspots: Asp69^HLA-E^, Asp162^HLA-E^, Arg68^HLA-E^, Gln72^HLA-E^, Asp163^CD94^, Glu164^CD94^, Phe114^CD94^, Arg171^CD94^, and Pro169^NKG2C^ (Supplementary Table S6 and Figure 6).

Furthermore, the peptide residues P5 and P6 formed the most important interactions for the ligand–receptor binding (Supplementary Table S5), while the P6 and P8 residues of the peptide could be considered as hotspots (Supplementary Table S6). The models including bound peptides B7 and G (**COM^−^_B7_** and **COM^+^_G_**, respectively) displayed energetically higher predispositions for the receptor–ligand binding compared to models **COM^~^_B27_** and **COM^−^_Cw7_** (Supplementary Table S7), which is in line with previous reports [25,26]. In addition, the **COM_apo_** model showed the least favorable ΔG_b_ for receptor–ligand binding compared to all other models (Supplementary Table S7).

## 3. Discussion

In our study, we generated five immune complexes of the HLA-E/β2m/NKG2C/CD94/peptide system to address the mechanistic details of the molecular recognition and signal transduction between the NKG2C/CD94 receptor on the NK cell and the HLA-E/β2m/peptide ligand at the atomistic level. Multi-microsecond all-atom MD simulations based on the available immune complex crystal structures [19] revealed no major conformational changes in any of the models possessing different NK cell activation abilities, demonstrating a “lock and key”-like interaction, analogous to the experimental data obtained for the NKG2A variant of the immune complex. Such an interaction, with no conformational changes taking place, is generally expected in receptor–ligand interactions of the innate immune system [9,19]. Further, the generated cross-correlation matrices revealed a nonameric peptide, the NKG2C protein, and the HLA-E_α2_ domain as the key components for the productive signal transduction. According to our models, the peptide that would enable the activation of the NK cell via this complex should cause a destabilization of the CD94 and NKG2C parts of the receptor, which could subsequently affect the communication between NKG2C and DAP12.

Furthermore, we revealed nine hotspots, which contribute the most to the binding free energy at the receptor–ligand complex interface. Two of them, namely, Asp162^HLA-E^ and Gln72^HLA-E^, have been reported previously [18]. Moreover, several hotspots and residues with large energy contributions to the ΔG_B_ disclosed here coincide with those known to play important roles in receptor–ligand binding in the parallel NKG2A immune system [9,17,18], such as, for example, Arg75^HLA-E^ and Asp162^HLA-E^ [18]. This suggests the existence of a joint cluster of the HLA-E residues that are energetically important for the interaction with both the NKG2A and NKG2C components of the NK receptors, as previously confirmed by mutagenesis studies [18]. The similarity between the activating and inhibitory receptors further extends to the observation that the energetically predominant portion of the receptor–ligand interactions is located at the CD94–HLA-E_α1_ site [18,19], which may leave less room for the HLA-E_α1_ domain movement. Flexibility pattern analysis indeed revealed the increased rigidity of the HLA-E_α1_ domain in all the models examined. The peptide-dependent influence on the flexibility of the neighboring HLA-E_α2_ domain further supports the concept of a peptide-mediated crosstalk between the receptor protein NKG2C and the ligand HLA-E_α2_ domain.

**Figure 7:**
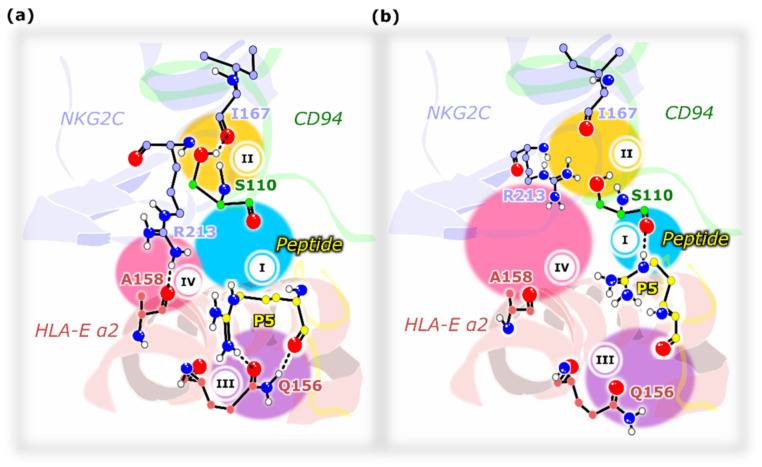
Schematic representation of the key residues putatively involved in activating the signal transduction for (**a**) model conferring the NK cell activation and (**b**) for the model with the absent NK cell activation. The Roman numerals show the possible sequence of events: (**I**) the P5–S110^CD94^ H-bond likely (**II**) abolishes the S110^CD94^–I167^NKG2C^ contact and (**III**) precludes the P5–Q156^HLA-E^ double interaction, which affects the positioning of the HLA-E_α2_ domain and thus Ala158^HLA-E^, (**IV**) allowing the formation of the R213^NKG2C^–A158^HLA-E^ H-bond. HLA-E—HLA class I histocompatibility antigen, alpha chain E, NKG2C—NKG2-C type II integral membrane protein, CD94—Natural killer cells antigen CD94.

Joining the results of the performed simulations together, key ligand–receptor interactions associated with two possible scenarios, taking place after the formation of the immune complex can be proposed (Figure 7). When the immune complex contains a peptide that leads to a successful NK cell activation (**COM^+^_G_**,), the (**I**) H-bond between the peptide P5 residue and Ser110^CD94^ is disrupted and thus not formed. This in turn enables the (**II**) formation of the Ser110^CD94^–Ile167^NKG2C^ H-bond as well as the (**III**) P5–Gln156^HLA-E^ double H-bond interaction, which is absent in the representative clusters of all three of the non-productive peptide-including models (**COM^~^_B27_**_,_ **COM^−^_B7_**, and **COM^−^_Cw7_**). The double H-bond between the P5 residue and Gln156^HLA-E^ could influence the positioning of the HLA-E_α2_ domain and the Ala158^HLA-E^ residue, consequently allowing the formation of the (**IV**) H-bond between the Arg213^NKG2C^–Ala158^HLA-E^ ligand–receptor residues. This H-bond network may ultimately influence the interactions between the NKG2C protein and the associated adaptor protein DAP12, leading to the NK cell activation. Indeed, a substitution of one of the key residues in this proposed mechanism, the peptide residue Arg^P5^, impairs the inhibitory signaling [27]. The Arg^P5^ residue was previously confirmed to be important for the overall affinity and the affinity differences between the NKG2A/CD94 and NKG2C/CD94 receptors [9]. Taken together, in this presented ligand–receptor model, the nonameric peptide acts similarly to a puppet hand that can pull the crucial strings via a specific hydrogen bonding network in a way that properly orchestrates the behavior of the NKG2C protein and the HLA-E_α2_ domain, ultimately leading to NK cell activation.

Besides the differences in the binding affinities between the NKG2C/CD94 (activating) and NKG2A/CD94 (inhibitory) receptors, their overall mechanism of signal transduction also differs. While the NKG2A/CD94 receptor inhibits the NK cells via its intracellular ITIM, the NKG2C/CD94 activates them via the adaptor protein DAP12, which carries the effector ITAM domain [7,8]. Given this difference, it is possible to speculate that the molecular recognition and mechanism of action carried out via these two receptors also differs. The NK cell activation is likely to be more tightly regulated, as the NK cell activation involving DAP12 depends on the proper orchestration of more proteins than the NK cell inhibition. This could also be one of the reasons for a more restricted batch of currently identified peptides that confer NK cell activation via the NKG2C/CD49 receptor [10].

Furthermore, we can also speculate about the reasons for the observed 6-fold difference in the affinity between the activating and inhibitory receptors, which may be related to the observed higher peptide specificity in the case of the NKG2C/CD94 activating receptor. The sequences of 165–168 (ASIL) or 167–170 (SIIS) residues that are supposedly responsible for the differences in the receptor–ligand binding affinity do not form direct contacts with the HLA-E/β2m/peptide ligand. They might play a role in the orientation of NKG2A(C)/CD94 heterodimer, which indirectly affects its interaction with the ligand [9]. Comparing the crystal structures of the NKG2A protein with the most representative conformation of the corresponding NKG2C homology model for the **COM^+^_G_** complex from our MD simulations, we observed that the ASIL sequence present in the NKG2C protein enables less hydrophobic contacts with its CD94 partner hydrophobic residues Val66^CD94^, Ile75^CD94^ Phe107^CD94^ and Met108^CD94^ (Supplementary Figure S6). With subsequent distance measurement and H-bond analysis, the presence of the Ser110^CD94^-Ile167^NKG**2C**^ interaction was identified during the productive MD run of the **COM^+^_G_** model (Supplementary Figure S2). Meanwhile, according to the crystallographic data, the corresponding Ser110^CD94^ forms a H-bond with Ser170^NKG2A^ in the NKG2A complex. (Supplementary Figure S6).

The Ser110^CD94^–Ile167^NKG2C^ H-bond is exclusively present in the **COM^+^_G_** immune complex for which the NK cell activation was experimentally confirmed [10,11]. Since the NKG2A protein contains more hydrophobic contacts between the CD94 and NKG2A receptors in the SIIS region part, along with the observed Ser110^CD94^–Ser170^NKG2A^ interaction connecting them, the identity of the peptide in the HLA-E/β2m/peptide ligand counterpart of the immune complex may not need to be so specifically tailored as in the case of the NKG2C system. As our **COM^+^_G_** simulations revealed, the Ser110^CD94^–Ile167^NKG2C^ H-bond formation depends on the incorporation of the favorable peptide, where we also speculate that P5 arginine plays a crucial role. Furthermore, Ile167^NKG2C^ is a conserved residue between NKG2C and NKG2A. The need for a very precise peptide to provide a key interaction, which would, according to our model, lead to NK cell activation, could further explain why the NK cell activation with the NKG2C-based immune complex is enabled with a more restricted set of peptides than inhibition via the NKG2A immune complex [10,11].

Finally, it should be noted that in our study, we utilized an all-atom molecular dynamics simulation as a computational microscope to investigate the atomistic machineries of these biomolecular systems. Despite rapid advances in the field and a general acceptance of this approach, some limitations are still present. The current force fields are still imperfect and furthermore, the molecular composition of the simulated biological systems is frequently too simplistically set up, leaving out different types of molecules. Furthermore, even the most powerful computational resources do not enable simulations up to timescales in which important biochemical events such as major conformational changes and binding events typically take place. In this respect, the development of enhanced sampling methods that cover the relevant conformational space as efficiently as possible, is essential to produce the most accurate description of the simulated biosystems [28].

## 4. Materials and Methods

### 4.1. Structural Models

We constructed five different models, all based on the available crystal structure of the extracellular domains of the human CD94/NKG2A immune receptor in a complex with the extracellular domain of the HLA-E (light and heavy chain) and the leader peptide of HLA class I histocompatibility antigen, alpha chain G, solved at 3.4 Å resolution (PDB ID 3CDG) [19]. Subsequently, the NKG2A portion of the complex was replaced by its homolog NKG2C protein, the 3D structure of which was modelled using the SWISS-MODEL server [29]. Given the high sequence identity between both the NKG2x proteins (~95%), we further assumed the same position of the NKG2C partner inside the immune complex. Therefore, we placed it in the complex by structural alignment with the NKG2A, which was originally present in the 3CDG crystal structure.

The first simulated model (i) **COM^+^_G_** consisted of HLA-E, β2m, NKG2C and CD94 proteins, and the G peptide (sequence: VMAPRTLFL). The remaining models differed from the initial **COM^+^_G_** model in the identity of the bound peptide. The second model (ii) **COM^~^_B27_** contained the B27 peptide (sequence: VTAPRTLLL, peptide taken from PDB ID 1KTL) [30]. The third model (iii) **COM^−^_B7_** included the B7 peptide (sequence: VMAPRTVLL, peptide taken from PDB ID 1KPR) [30] and the forth (iv) **COM^−^_Cw7_** consisted of the signal sequence of the HLA-Cw7 molecule (peptide taken from PDB ID 3BZF) [31]. In the last model (v) **COM_apo_** the bound peptide was removed.

### 4.2. Molecular Dynamics (MD) Simulations

When preparing the constructed systems for the simulations, the protonation states of the ionizable residues were assigned by the PDB2PQR web tool at pH 7 [32]. Histidines were protonated either at Nε2 or Nδ1 atoms, or at both positions. Carboxylic amino acids were in their common deprotonated states. Next, the protein complex was embedded in a 10 Å layer of TIP3P water molecules [33] resulting in a box of 126.4 × 131.5 × 118.9 Å^3^. Together with the water molecules and 18 Na^+^ counterions, the systems consisted of approximately 193,540 atoms. Model topologies were built and disulfide bonds were constructed with *tleap* module of Ambertools 18 [34].

The initial energy minimization of each system was followed by a gradual heating in two successive steps: 0–100 K over 5 ps in the first step and 100–303 K over the next 120 ps in the second step. Positional restraints of 200 kcal/mol Å^2^ and 100 kcal/mol Å^2^ for all heavy atoms were applied, respectively. The restraints were then removed and a 10 ns of isothermal-isobaric ensemble (NPT) was performed, using a Berendsen barostat for the pressure control (1 bar) [35]. Next, a 1.2 μs production MD run using canonical ensemble (NVT) with periodic boundary conditions was conducted for each model, resulting in a total simulation time of ~6 µs. During the MD simulations, a temperature control (T = 303 K) with a collision frequency of 1 ps^−1^ was performed by applying the Langevin thermostat [36]. The SHAKE algorithm [37] was used to constrain the hydrogen bonds, and the particle mesh Ewald method [38] with a cutoff of 10 Å was utilized to account for the long-range electrostatic interactions. An integration time step of 2 fs was used for all MD runs.

VMD [39] and PyMol [40] software were used to visualize the obtained MD trajectories. The *cpptraj* module in Ambertools 18 [34] and Gromacs 2016 [21] suite were employed to perform the trajectory analyses, including the root-mean-square fluctuations (RMSF) and the calculation of the cross-correlation matrices. For these analyses, the water and counterion-stripped trajectories were used considering 3334 frames, corresponding to the last 1 μs of each performed MD production run.

The occurrences of hydrogen (H)-bonds were identified by the *cpptraj* module in Ambertools 18 [34], using a distance cutoff of 3.0 Å along with an angular cutoff of 135°. The same module was also used for the cluster analysis to assess the structural populations. For the latter, a hierarchical agglomerative approach, a distance cutoff of ~2 Å, and a distance metric of the mass-weighted root-mean-square deviation (RMSD) of the backbone atoms were employed [41].

### 4.3. Cross-Correlation Matrix and Correlation Scores

The cross-correlation matrix allows the disclosure of possible relationships between different components of the simulated complex. The matrix is based on the Pearson’s correlation coefficients (*CCij*) and quantifies the (anti-)correlated movements between a pair of residues along the MD trajectory. *CCij* values can range from −1 to +1, where 0 represents no correlation between two residues, −1 indicates fully anti-correlated, and +1 corresponds to a fully correlated motion.

The covariance matrices were formed from the atomic position vectors. By using the RMS-fit to a reference structure, which was the averaged structure from the MD production run, only the internal dynamics of the complex were captured, while the rotational and translational motions were removed [42,43,44]. The obtained covariance matrices were then adopted to calculate the cross-correlation matrices and the normalized covariance matrices using the *cpptraj* module in Ambertools 18 [34]. To obtain a simplified version of the *CCij* matrix that more clearly illustrated the relationships between individual proteins and/or (sub)domains of the multicomponent immune complex, the correlations for each protein–domain pair were assessed by summing the correlation scores (*CSs*) between each pair and all the others. Next, a correlation density for each area was obtained by summing the *CSs* of a protein–domain pair, which was then divided by a product of a number of residues belonging to that protein–domain pair [45,46].

### 4.4. Energy Calculations

The binding free energies, first between the complex HLA-E/β2m/NKG2C/CD94 and the bound peptide pairs, and, second, between the receptor NKG2C/CD94 and the ligand HLA-E/β2m/peptide pairs were calculated using the molecular mechanics-generalized born surface area (MM-GBSA) method [20] and the Amber18 code [34]. MM-GBSA calculations were performed for 100 equally spaced frames from each MD trajectory taken from the production MD run interval between 600 and 900 ns. The igb flag value was set to 5, and a salt concentration of 0.1 M was used. Pairwise and per-residue decomposition energy analyses were performed for both used divisions of the system. The conformational entropic contribution of the free energy was not included in the calculations because it was previously suggested that this term does not improve the quality of the results when using the MM-G(P)BSA [47]. The interaction energies were also calculated by the gmx energy module in Gromacs2016 [21] software package on the equilibrated part of the trajectories, considering the last 1 μs of the MD production run.

## 5. Conclusions

In summary, we performed and analyzed several multi-microsecond molecular dynamics simulations (MD) that harvested novel information for the ongoing quest to understand the atomistic details that are critical for the successful molecular recognition between the HLA-E/β2m/peptide ligand and the activating NKG2C/CD94 receptor and subsequent signal transduction in the fundamental immune process event of the natural killer (NK) cell action. The findings obtained in this study suggest that the peptide acts like a puppet hand that can pull the crucial strings via a specific hydrogen bonding network to orchestrate the actions of the NKG2C and HLA-E_α2_ domains leading to NK cell activation. This information may provide the necessary foundation for the targeted design of new biochemical and structural studies to further decipher the mechanistic details of the deficiently understood HLA-E/β2m/peptide/NKG2C/CD94 immune complex.

## Figures and Tables

**Table 1:** Generated models of the HLA-E/peptide/β2m/NKG2C/CD94 immune complexes utilized in the MD simulations with denoted peptide sequences, NK cell activation ability [10,11], and assigned model number.

Model	Peptide	Peptide Sequence	NK Cell Activation	Model Number
**COM^+^_G_**	G	VMAPRTLFL	**yes (+)**	i
**COM^~^_B27_**	B27	VTAPRTLLL	**inconclusive (~)**	ii
**COM^−^_B7_**	B7	VMAPRTVLL	**no (^−^)**	iii
**COM^−^_Cw7_**	Cw7	VMAPRALLL	**no (^−^)**	iv
**COM_apo_**	/	/	**N/A**	v

**Table 2:** H-bonds previously identified in the HLA-E/β2m/peptide/NKG2A/CD94 immune complex as calculated by *cpptraj* module in Ambertools for the simulated NKG2C-based immune complexes.

**COM^+^_G_**			**COM^~^_B27_**		
**Donor**	**Acceptor**	**%**	**Donor**	**Acceptor**	**%**
THR_6@O^P6^	GLN_112@NE2^CD94^	76	THR_6@O^P6^	GLN_112@NE2^CD94^	33
GLU_152@OE1^HLA-E^	ARG_5@NH1^P5^	48	GLU_152@OE1^HLA-E^	ARG_5@NH1^P5^	25
SER_110@O^CD94^	ARG_5@NH2^P5^	10	SER_110@O^CD94^	ARG_5@NH1^P5^	46
**COM^−^_B7_**			**COM^−^_Cw7_**		
**Donor**	**Acceptor**	**%**	**Donor**	**Acceptor**	**%**
THR_6@O^P6^	GLN_112@NE2^CD94^	45	ALA_6@O^P6^	GLN_112@NE2^CD94^	74
GLU_152@OE1^HLA-E^	ARG_5@NH2^P5^	62	GLU_152@OE1^HLA-E^	ARG_5@NH1^P5^	49
SER_110@O^CD94^	ARG_5@NH2^P5^	47	SER_110@O^CD94^	ARG_5@NH2^P5^	46

## Data Availability

All molecular simulations, analysis and visualization were performed with the broadly used programs freely available for academic institutions: Gromacs 2016, Amber, AmberTools 18, VMD 1.9.3 and PyMOL 2.0. Starting structures were obtained from the Protein Data Bank (PDB) public database. All procedures and workflows are described in the Methods Section.

## Supplementary Materials

The following are available online at https://www.mdpi.com/article/10.3390/ijms22136670/s1. Supplementary_file_1.pdf: Supplementary Figures and Tables; Supplementary_file_2.mp4: Supplementary Movie.
