# Supplementary material for: Nonameric Peptide Orchestrates Signal Transduction in the Activating HLA-E/NKG2C/CD94 Immune Complex as Revealed by All-Atom Simulations
Source: Int J Mol Sci. 2021 Jun 22;22(13):6670. doi: 10.3390/ijms22136670 (PMC8268078; doi:10.3390/ijms22136670)
Supplement: Supplementary file 1 [file ijms-22-06670-s001.zip › Supplementary_file_1.pdf]

Supplementary Materials

Nonameric Peptide Orchestrates Signal Transduction in the Activating HLA-E/NKG2C/CD94 Immune Complex as Revealed by All-atom Simulations

Eva Prašnikar[†,‡], Andrej Perdih[†,&,*], Jure Borišek[†,*]

[†]National Institute of Chemistry, Hajdrihova 19, 1000, Ljubljana, Slovenia
[‡]Graduate School of Biomedicine, Faculty of Medicine, University of Ljubljana, Vrazov trg 2, 1000 Ljubljana, Slovenia
[&]Faculty of Pharmacy, University of Ljubljana, Aškerčeva 7, 1000 Ljubljana Slovenia

Corresponding authors*:
E-mail: andrej.perdih@ki.si and jure.borisek@ki.si

Table of contents

Supplementary figures 3
Figure S1 3
Figure S2 4
Figure S3 5
Figure S4 6
Figure S5 7
Figure S6 8
Supplementary tables 9
Table S1 9
Table S2 10
Table S3 11
Table S4 12
Table S5 13
Table S6 14
Table S7 15
Supplementary movie 16
Movie S1. 16
Supplementary references 17

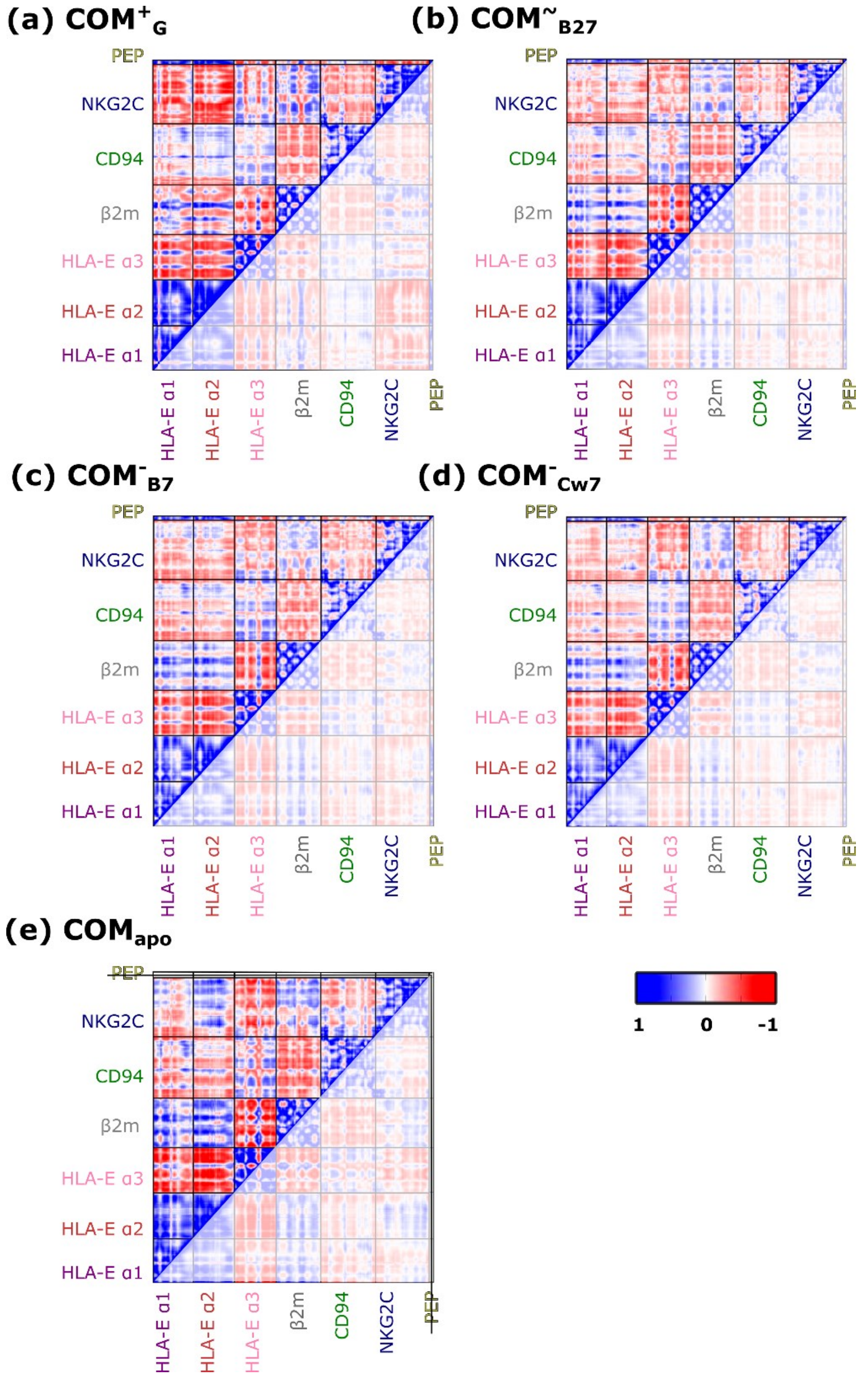

Figure S1. Cooperative motion underlying the functional dynamics of the models (a) **COM^{+}$_{G}$**, (b) **COM$^{\sim}$$_{B27}$**, (c) **COM^{-}$_{B7}$**, (d) **COM^{-}$_{Cw7}$**, and (e) **COM$_{apo}$**. Per-residue Pearson's coefficients (*CCs*) cross-correlation matrix was derived from the mass-weighted covariance matrix calculated over the last 1 µs of the classical molecular dynamics trajectories. CCs values range from -1 (red, anticorrelated motions) to +1 (blue, correlated motions). For the cross-correlation matrices calculation HLA-E α1, α2, and α3 domains, β2m, CD94, NKG2C and peptide are considered.

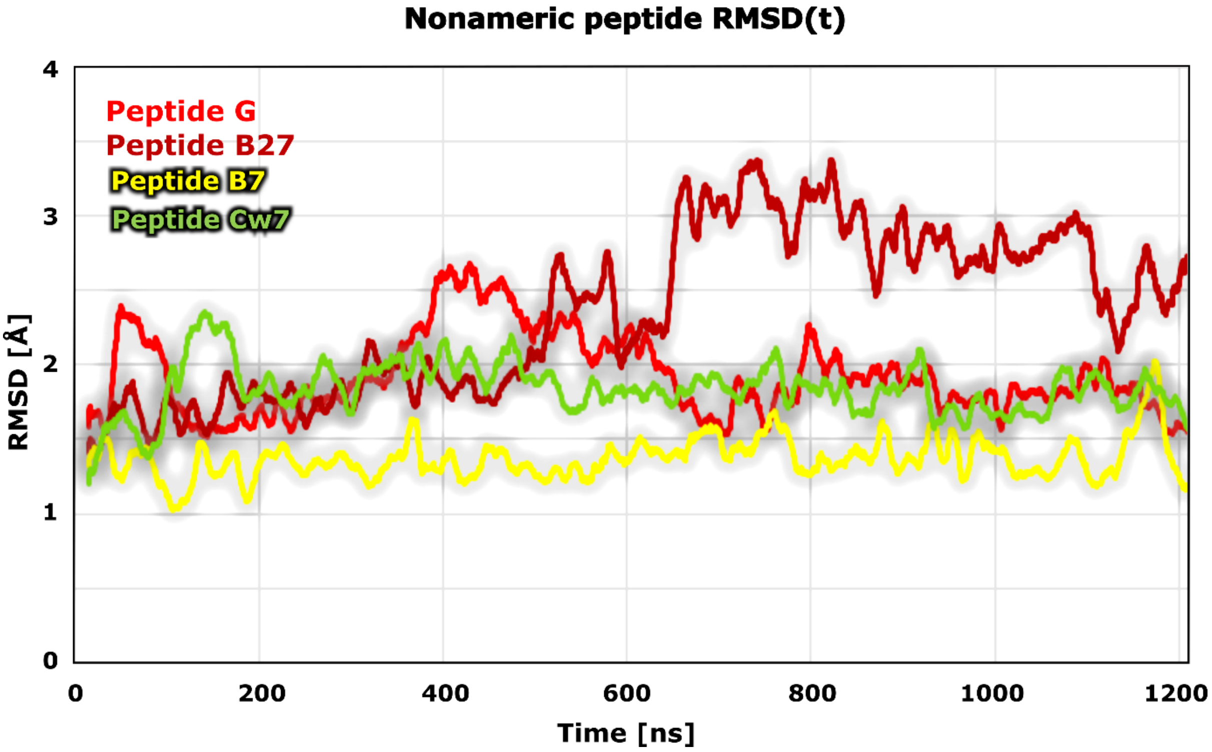

Figure S2. Nonameric peptide's root mean square deviation (RMSD) for models **COM^{+}$_{G}$** (G peptide), **COM$^{\sim}$$_{B27}$** (B27 peptide), **COM^{-}$_{B7}$** (B7 peptide), and **COM^{-}$_{Cw7}$** (Cw7 peptide). The moving average with interval 20 was used in data processing.

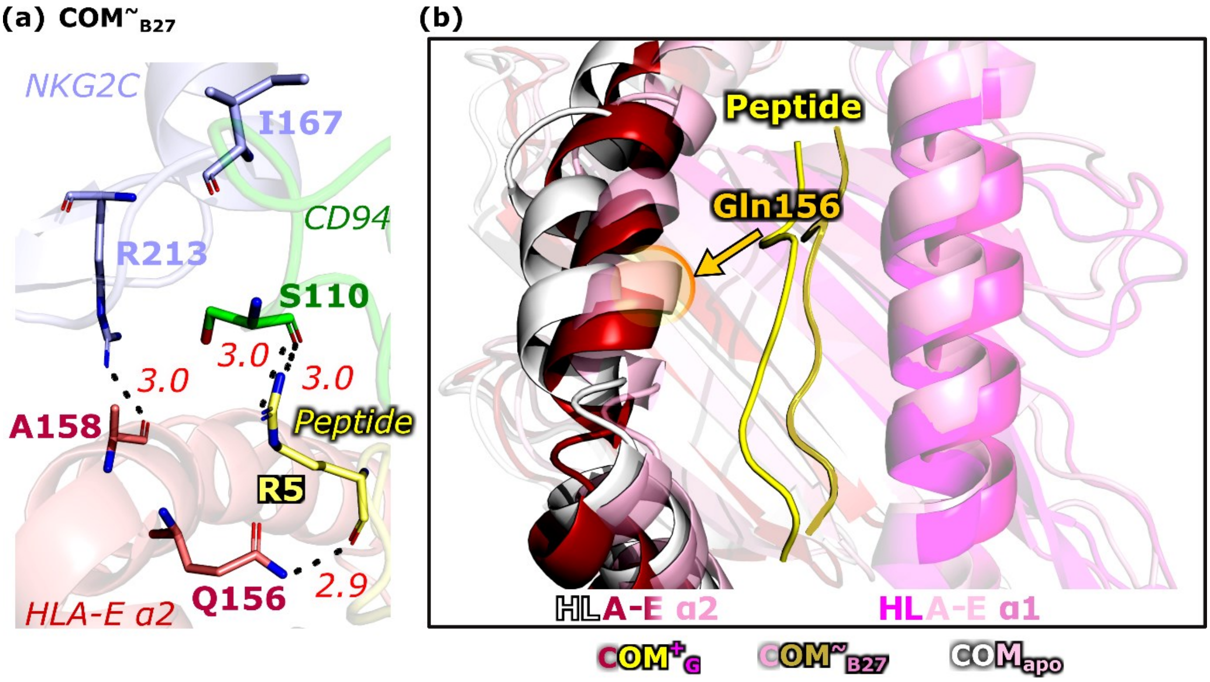

Figure S3. Key H-bond interaction differences observed between the simulated models. (a) Hydrogen bonds between putative key residues S110, Q156, A158 and R5 in signal transduction for **COM˜$_{\mathbf{B27}}$** model. (b) The alignment of the HLA-E$_{\alpha2}$ region for the **COM$^{+}_{\mathbf{G}}$**, **COM˜$_{\mathbf{B27}}$** and **COM$_{\mathbf{apo}}$** models with highlighted position of Gln156$^{\text{HLA-E}}$. Data is derived from most representative cluster of the last 500 ns of simulation time.

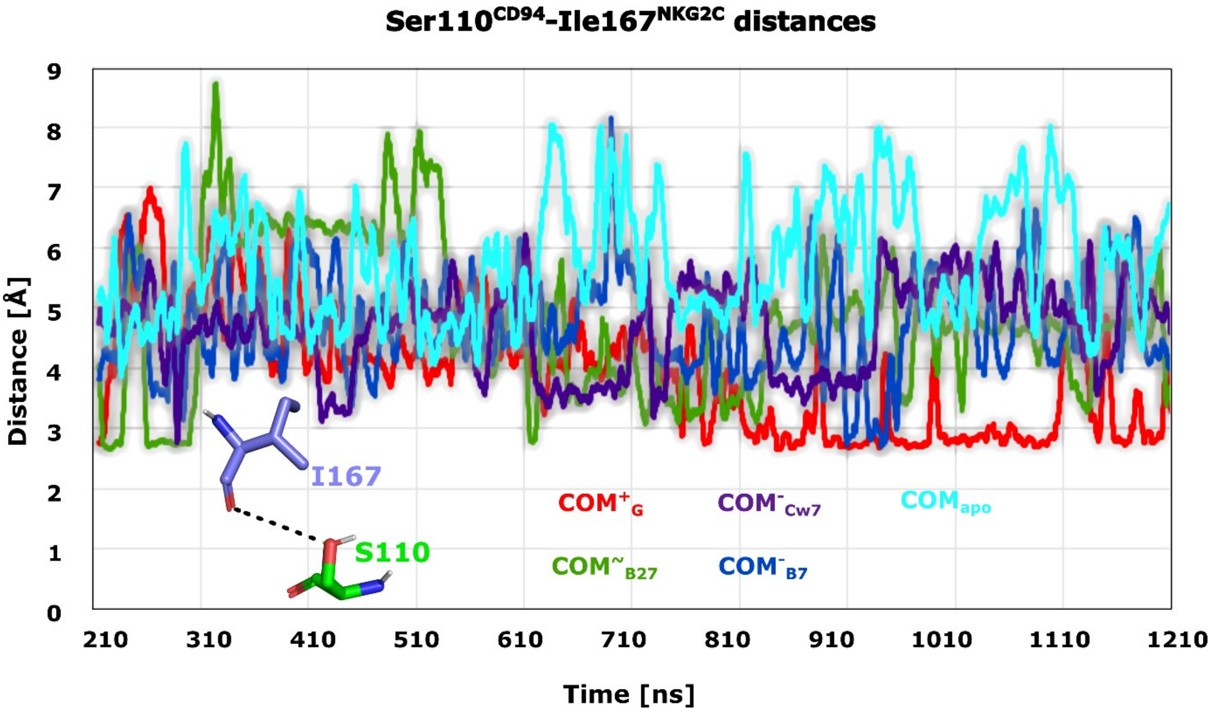

Figure S4. Graph of Ser110^{CD94} (@OG) and Ile167^{NKG2C} (@O) hydrogen bond distances as a function of time for models **COM$^{+}_{G}$** (red), **COM$^{\sim}_{B27}$** (green), **COM$^{-}_{B7}$** (blue), **COM$^{-}_{Cw7}$** (violet)**,** and **COM$_{apo}$** (cyan). The moving average with interval 20 was used in data processing. The establishment of the Ser110^{CD94}-Ile167^{NKG2C} hydrogen bond occurs only for the **$_{2C}$COM$^{+}_{G}$** (red) model in the last half of the trajectory.

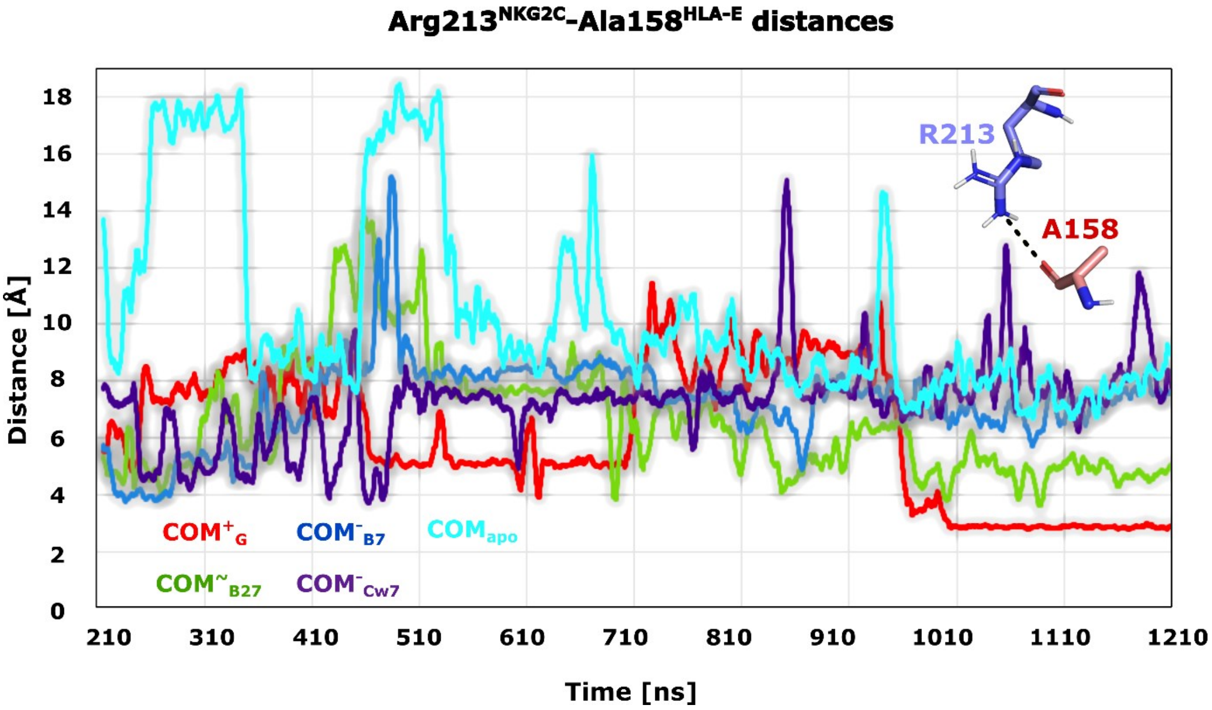

Figure S5. Graph of Arg213^{NKG2C} (@NH2)-Ala158^{HLA-E} (@O) hydrogen bond distances as a function of time for models **COM$^{+}_{G}$** (red), **COM$^{\sim}_{B27}$** (green), **COM$^{-}_{B7}$** (blue), **COM$^{-}_{Cw7}$** (violet)**,** and **COM$_{apo}$** (cyan)**.** The moving average with interval 20 was used in data processing. The establishment of the Arg213^{NKG2C}-Ala158^{HLA-E} hydrogen bond is more evident only for the **$_{2C}$COM$^{+}_{G}$** (red) model in the last part of the trajectory.

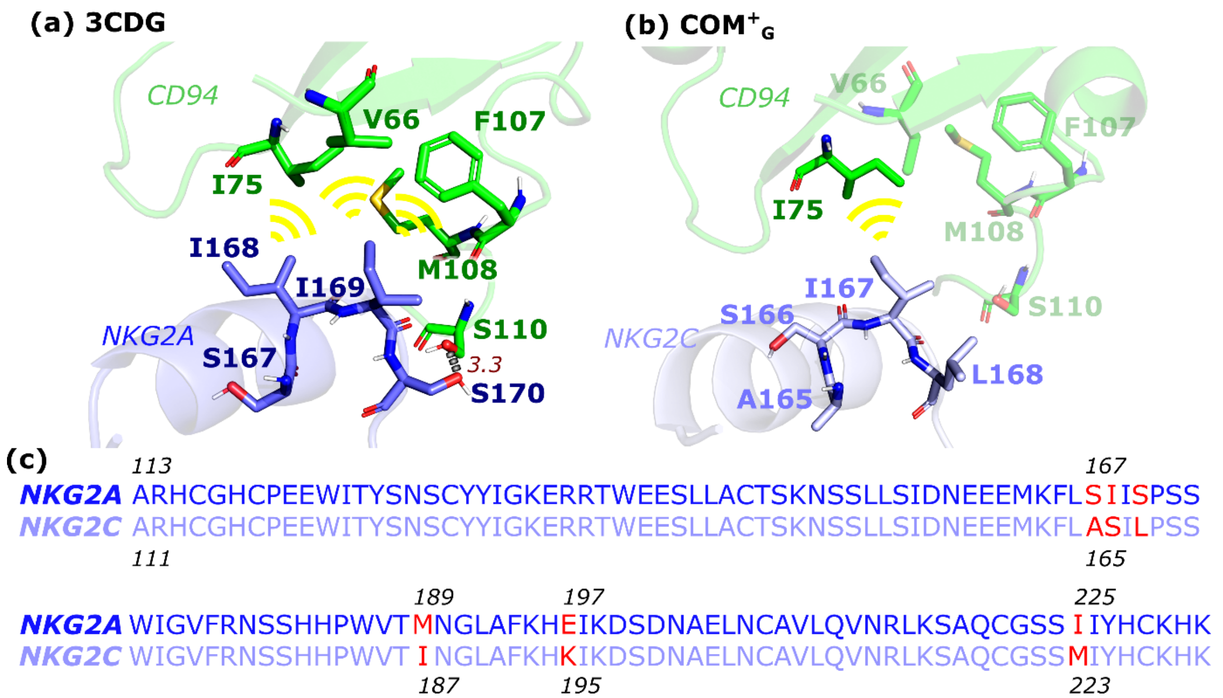

(c)

```
        113                                                                    167
NKG2A   ARHCGHCPEEWITYSNSCYYIGKERRTWEESLLACTSKNSSLLSIDNEEEMKFLSIISPSS
NKG2C   ARHCGHCPEEWITYSNSCYYIGKERRTWEESLLACTSKNSSLLSIDNEEEMKFLASILPSS
        111                                                                    165

                           189        197                                   225
NKG2A   WIGVFRNSSHHPWVTMNGLAFKHEIKDSDNAELNCAVLQVNRLKSAQCGSSIIYHCKHK
NKG2C   WIGVFRNSSHHPWVTINGLAFKHKIKDSDNAELNCAVLQVNRLKSAQCGSSMIYHCKHK
                           187        195                                   223
```

Figure S6. Comparison of NKG2C (light blue) and NKG2A (blue) interactions with CD94 (green) in the ASIL/SIIS region for (a) the crystal structure of NKG2A/CD94/HLA-E/β2m/peptide immune complex (PDB ID 3CDG) and (b) the most representative cluster of the **COM^{+}$_{G}$** immune complex. Only polar hydrogens are displayed. (c) The alignment of the NKG2A and NKG2C sequences with amino acid differences marked with red. Interacting residues are depicted in licorice and transparency is set when interaction is not repeated in **COM^{+}$_{G}$** (residues V66, F107, M108, and S110).

Supplementary tables

Table S1. List of hydrogen bonds identified in the experimentally determined HLA-E/β2m/peptide/NKG2A/CD94 immune complex[1] that are also found in the most representative cluster obtained from the MD simulation trajectories.

Interaction	$_{2C}COM^{+}_{G}$	$_{2C}COM^{\sim}_{B27}$	$_{2C}COM^{-}_{B7}$	$_{2C}COM^{-}_{Cw7}$
P6-Gln112^{CD94}	•			
P5-Glu152^{HLA-E}	•	•	•	•
P5-Ser110^{CD94}			•	•

Table S2. Molecular mechanics-generalized born surface area (MM-GBSA)[2] binding free energies (ΔG_b) between peptide and the rest of the immune complex (HLA-E/β2m/NKG2C/CD94) and pair-wise free energies decomposition (kcal/mol) calculated over time interval between 600 and 900 ns of the production MD trajectory for **COM$^{+}_{G}$**, **COM$^{\sim}_{B27}$**, **COM$^{-}_{B7}$**, and **COM$^{-}_{Cw7}$** models. In the table are gathered either 10 major ΔGb energies or energies with the lower cutoff of -0.5 kcal/mol for interactions between peptide and the rest of the immune complex. Interactions in bold represent contacts previously reported for the immune complex of NKG2A variant,[1] meanwhile orange marks the engagement between residues with putative key role in signal transduction.

**COM$^{+}_{G}$ ΔG_b -111.6 ± 10.5 (kcal/mol)**		
**Res 1**	**Res 2**	**ΔG_b total [kcal/mol]**
CD94		
**Gln112**	**Thr^{P6}**	**-4.0 ± 0.5**
**Gln112**	**Phe^{P8}**	**-3.3 ± 0.7**
**Asn158**	**Phe^{P8}**	**-1.7 ± 0.8**
**Asn160**	**Phe^{P8}**	**-1.2 ± 1.0**
**Gln112**	**Arg^{P5}**	**-1.1 ± 1.0**
**Asn156**	**Phe^{P8}**	**-0.9 ± 0.3**
Ser110	Arg^{P5}	-0.9 ± 1.4
**Phe114**	**Phe^{P8}**	**-0.7 ± 0.2**
Gln113	Arg^{P5}	-0.5 ± 0.3
NKG2C		
**Pro169**	**Arg^{P5}**	**-0.9 ± 0.4**
Val211	Pro^{P4}	-0.8 ± 0.3
Val211	Arg^{P5}	-0.5 ± 0.4
HLA-E		
Glu63	Val^{P1}	-25.2 ± 3.2
**Glu152**	**Arg^{P5}**	**-18.5 ± 2.3**
Lys146	Leu^{P9}	-13.7 ± 4.7
Gln156	Arg^{P5}	-9.6 ± 0.8
Ser143	Leu^{P9}	-6.7 ± 1.7
Tyr171	Val^{P1}	-5.7 ± 1.1
Tyr84	Leu^{P9}	-5.4 ± 1.9
Tyr7	Val^{P1}	-4.7 ± 1.7
Trp167	Val^{P1}	-4.3 ± 1.4
Thr80	Leu^{P9}	-4.1 ± 1.9

**COM$^{\sim}_{B27}$ ΔG_b -80.9 ± 8.3 (kcal/mol)**		
**Res 1**	**Res 2**	**ΔG_b total [kcal/mol]**
CD94		
**Ser110**	**Arg^{P5}**	**-7.3 ± 1.2**
**Gln112**	**Arg^{P5}**	**-3.4 ± 1.3**
**Gln112**	**Thr^{P6}**	**-3.2 ± 0.9**
**Gln112**	**Leu^{P8}**	**-1.8 ± 0.7**
**Asn160**	**Leu^{P8}**	**-0.9 ± 0.4**
**Asn156**	**Leu^{P8}**	**-0.8 ± 0.3**
**Asn158**	**Leu^{P8}**	**-0.8 ± 0.5**
**Phe114**	**Leu^{P8}**	**-0.7 ± 0.3**
Gln112	Pro^{P4}	-0.5 ± 0.8
HLA-E		
**Glu152**	**Arg^{P5}**	**-17.8 ± 2.4**
Glu63	Val^{P1}	-14.2 ± 2.8
Lys146	Leu^{P9}	-13.5 ± 4.1
Glu63	Thr^{P2}	-9.5 ± 2.1
Ser428	Arg^{P5}	-7.3 ± 1.2
Ser143	Leu^{P9}	-5.6 ± 2.6
Tyr84	Leu^{P9}	-4.5 ± 2.6
Gln156	Arg^{P5}	-4.2 ± 0.8
Trp97	Thr^{P6}	-3.6 ± 0.8
Gln430	Arg^{P5}	-3.4 ± 1.3

**COM$^{-}_{B7}$ ΔG_b -98.3 ± 8.2 (kcal/mol)**		
**Res 1**	**Res 2**	**ΔG_b total [kcal/mol]**
CD94		
**Ser110**	**Arg^{P5}**	**-5.7 ± 1.6**
**Gln112**	**Thr^{P6}**	**-3.9 ± 0.5**
**Gln112**	**Arg^{P5}**	**-2.3 ± 1.2**
**Gln112**	**Leu^{P8}**	**-1.9 ± 0 .5**
**Asn158**	**Leu^{P8}**	**-1.0 ± 0.4**
**Asn160**	**Leu^{P8}**	**-0.8 ± 0.3**
NKG2C		
Val211	Pro^{P4}	-1.3 ± 0.4
**Pro169**	**Arg^{P5}**	**-1.2 ± 0.3**
Val211	Arg^{P5}	-0.9 ± 0.3
HLA-E		
Glu63	Val^{P1}	-20.4 ± 3.9
**Glu152**	**Arg^{P5}**	**-18.1 ± 2.8**
Lys146	Leu^{P9}	-16.0 ± 4.0
Ser143	Leu^{P9}	-7.3 ± 1.0
Tyr84	Leu^{P9}	-6.2 ± 1.1
Tyr7	Val^{P1}	-5.8 ± 1.6
Tyr171	Val^{P1}	-5.4 ± 1.1
Trp167	Val^{P1}	-5.0 ± 1.5
Thr80	Leu^{P9}	-4.9 ± 2.0
Gln156	Arg^{P5}	-3.8 ± 0.9

**COM$^{-}_{Cw7}$ ΔG_b -97.1 ± 9.5 (kcal/mol)**		
**Res 1**	**Res 2**	**ΔG_b total [kcal/mol]**
CD94		
**Ser110**	**Arg^{P5}**	**-3.9 ± 1.8**
**Gln112**	**Ala^{P6}**	**-3.8 ± 1.2**
**Gln112**	**Arg^{P5}**	**-2.5 ± 1.6**
**Gln112**	**Leu^{P8}**	**-1.7 ± 0.6**
**Asn158**	**Leu^{P8}**	**-0.8 ± 0.4**
Gln113	Arg^{P5}	-0.7 ± 0.4
**Gln112**	**Leu^{P7}**	**-0.5 ± 0.6**
**Asn160**	**Leu^{P8}**	**-0.4 ± 0.3**
NKG2C		
**Pro169**	**Arg^{P5}**	**-1.6 ± 0.2**
HLA-E		
Glu63	Val^{P1}	-23.9 ± 3.4
**Glu152**	**Arg^{P5}**	**-19.3 ± 2.2**
Lys146	Leu^{P9}	-12.5 ± 5.6
Ser143	Leu^{P9}	-6.4 ± 1.9
Tyr171	Val^{P1}	-5.5 ± 1.1
Tyr7	Val^{P1}	-5.4 ± 1.7
Tyr84	Leu^{P9}	-4.8 ± 2.4
Glu63	Met^{P2}	-4.5 ± 1.5
Thr80	Leu^{P9}	-3.7 ± 1.9
His99	Met^{P2}	-3.5 ± 0.4

Table S3. Molecular mechanics-generalized born surface area (MM-GBSA)[2] binding free energies (ΔG_b) between peptide and HLA-E/β2m/NKG2C/CD94 and per-residue free energy decomposition (kcal/mol) calculated over time interval between 600 and 900 ns of the MD trajectory of the production run for **COM^{+}_{G}**, **$COM^{\sim}_{B27}$**, **COM^{-}_{B7},** and **COM^{-}_{Cw7}** models. In the table are listed energies for peptide residues. Interactions in bold represent primary anchor positions of the peptide in its binding site.

**COM^{+}_{G} ΔG_b -111.6 ± 10.5 kcal/mol**		**$COM^{\sim}_{B27}$ ΔG_b -80.9 ± 8.3 kcal/mol**		**COM^{-}_{B7} ΔG_b -98.3 ± 8.2 kcal/mol**		**COM^{-}_{Cw7} ΔG_b -97.1 ± 9.5 kcal/mol**	
**Residue**	**ΔG_b total**	**Residue**	**ΔG_b total**	**Residue**	**ΔG_b total**	**Residue**	**ΔG_b total**
**Met^{P2}**	-11.4 ± 1.2	**Leu^{P9}**	-7.7 ± 2.5	**Leu^{P9}**	-10.0 ± 2.6	**Met^{P2}**	-11.7 ± 1.2
Phe^{P8}	-9.0 ± 1.1	Arg^{P5}	-6.6 ± 1.7	**Met^{P2}**	-9.5 ± 1.5	**Leu^{P9}**	-8.2 ± 3.0
**Leu^{P9}**	-8.4 ± 2.7	Leu^{P8}	-6.1 ± 0.9	Leu^{P8}	-5.2 ± 1.0	Leu^{P7}	-5.9 ± 0.9
Leu^{P7}	-5.5 ± 0.9	**Thr^{P2}**	-6.0 ± 1.8	Val^{P7}	-4.0 ± 1.0	Leu^{P8}	-5.1 ± 1.0
Thr^{P6}	-3.8 ± 1.0	Leu^{P7}	-6.0 ± 0.8	Arg^{P5}	-3.9 ± 2.2	Ala^{P6}	-3.9 ± 1.2
Arg^{P5}	-3.6 ± 1.9	Thr^{P6}	-2.0 ± 1.5	Thr^{P6}	-3.3 ± 0.9	Ala^{P3}	-3.1 ± 0.4
Ala^{P3}	-2.0 ± 0.5	Pro^{P4}	-1.9 ± 0.6	Ala^{P3}	-2.1 ± 0.6	Pro^{P4}	-1.3 ± 0.3
Pro^{P4}	-1.6 ± 0.4	Ala^{P3}	-1.7 ± 0.7	Pro^{P4}	-1.9 ± 0.6	Arg^{P5}	-1.3 ± 2.0
Val^{P1}	1.4 ± 2.2	Val^{P1}	6.9 ± 2.3	Val^{P1}	2.4 ± 2.2	Val^{P1}	1.0 ± 2.1

Table S4. Comparison of the binding free energies (ΔG_b) and interaction energies between nonameric peptide and HLA-E/β2m/NKG2C/CD94 calculated by a) Molecular mechanics-generalized born surface area (MM-GBSA)[2] (kcal/mol) and by b) gmx energy module of the Gromacs2016[3] software package (kJ/mol) approaches.

a)

Model	VDW [kcal/mol]	EEL [kcal/mol]	EGB [kcal/mol]	ESURF [kcal/mol]	ΔG_b [kcal/mol]
COM^{+}_{G}	-110.0 ± 5.6	-550.6 ± 32.7	566.0 ± 26.3	-17.0 ± 0.5	-111.6 ± 10.5
$COM^{\sim}_{B27}$	-93.4 ± 5.9	-491.7 ± 27.3	519.6 ± 23.1	-15.4 ± 0.5	-80.9 ± 8.3
COM^{-}_{B7}	-105.0 ± 0.5	-530.4 ± 2.4	553.6 ± 1.9	-16.6 ± 0.0	-98.3 ± 0.8
COM^{-}_{Cw7}	-98.6 ± 0.5	-516.8 ± 3.4	533.4 ± 2.8	-15.2 ± 0.0	-97.1 ± 0.9

b)

Model	VDW [kJ/mol]	EEL [kJ/mol]	ΔG_b [kJ/mol]	ΔG_b [kcal/mol]
COM^{+}_{G}	-732.7 ± 60.5	-373.9 ± 23.7	-1106.6 ± 84.2	-264.3 ± 20.1
$COM^{\sim}_{B27}$	-654.9 ± 67.3	-321.0 ± 25.1	-975.9 ± 92.4	-233.1 ± 22.1
COM^{-}_{B7}	-698.0 ± 57.3	-362.6 ± 23.3	-1060.6 ± 80.6	-253.3 ± 19.2
COM^{-}_{Cw7}	-667.5 ± 70.2	-329.2 ± 23.0	-996.7 ± 93.3	-238.1 ± 22.3

Table S5. Molecular mechanics-generalized born surface area (MM-GBSA)[2] binding free energies (ΔG_b) between receptor (NKG2**C**/CD94) and ligand (HLA-E/β2m/peptide) and pair-wise free energy decomposition (kcal/mol) calculated over time interval between 600 and 900 ns of the MD trajectory in the production run for **COM$^{+}_{G}$**, **COM$^{\sim}_{B27}$**, **COM$^{-}_{B7}$,** and **COM$^{-}_{Cw7}$**. In the table are listed top 15 ligand-receptor interactions with highest energies.

**COM$^{+}_{G}$ ΔG_b -57.0 ± 15.3 kcal/mol**		
**ligand**	**receptor**	**ΔG_b total**
HLA-E		
Asp69^{HLA-E}	Arg171^{CD94}	-17.6 ± 1.6
Arg75^{HLA-E}	Asp163^{CD94}	-16.0 ± 2.6
Arg68^{HLA-E}	Glu162^{CD94}	-13.9 ± 4.9
Asp162^{HLA-E}	Arg213^{NKG2C}	-11.7 ± 6.6
Gln72^{HLA-E}	Glu164^{CD94}	-8.4 ± 2.3
Arg68^{HLA-E}	Asp168^{CD94}	-8.4 ± 5.3
Glu154^{HLA-E}	Arg135^{NKG2C}	-6.7 ± 6.1
Glu166^{HLA-E}	Arg213^{NKG2C}	-6.1 ± 7.2
Asp162^{HLA-E}	Lys215^{NKG2C}	-6.0 ± 4.5
Asp162^{HLA-E}	Lys197^{NKG2C}	-5.4 ± 5.2
Glu166^{HLA-E}	Lys197^{NKG2C}	-4.6 ± 4.6
Glu154^{HLA-E}	Arg157^{HLA-E}	-3.6 ± 1.2
Arg75^{HLA-E}	Glu164^{CD94}	-3.3 ± 2.0
Peptide		
Thr^{P6}	Gln112^{CD94}	-3.9 ± 0.5
Phe^{P8}	Gln112^{CD94}	-3.3 ± 0.7

**COM$^{\sim}_{B27}$ ΔG_b -52.3 ± 11.9 kcal/mol**		
**ligand**	**receptor**	**ΔG_b total**
HLA-E		
Arg79^{HLA-E}	Asp163^{CD94}	-16.3 ± 2.6
Asp69^{HLA-E}	Arg171^{CD94}	-15.4 ± 1.9
Asp162^{HLA-E}	Arg213^{NKG2C}	-14.6 ± 4.5
Arg75^{HLA-E}	Glu164^{CD94}	-9.7 ± 6.1
Arg75^{HLA-E}	Asp163^{CD94}	-9.6 ± 6.4
Arg68^{HLA-E}	Asp168^{CD94}	-8.1 ± 4.6
Asp162^{HLA-E}	Lys215^{NKG2C}	-7.7 ± 5.5
Gln72^{HLA-E}	Glu164^{CD94}	-6.4 ± 2.2
Asp162^{HLA-E}	Lys197^{NKG2C}	-6.3 ± 4.5
Glu152^{HLA-E}	Arg^{P5}	-3.2 ± 0.7
His155^{HLA-E}	Ser170^{NKG2C}	-3.0 ± 0.5
His155^{HLA-E}	Pro169^{NKG2C}	-2.9 ± 0.4
Peptide		
Arg^{P5}	Ser110^{CD94}	-7.3 ± 1.7
Arg^{P5}	Gln112^{CD94}	-3.5 ± 1.4
Thr^{P6}	Gln112^{CD94}	-3.2 ± 1.0

**COM$^{-}_{B7}$ ΔG_b -55.1 ± 13.1 kcal/mol**		
**ligand**	**receptor**	**ΔG_b total**
HLA-E		
Arg68^{HLA-E}	Glu164^{CD94}	-16.0 ± 7.6
Asp69^{HLA-E}	Arg171^{CD94}	-15.9 ± 1.8
Glu166^{HLA-E}	Arg213^{NKG2C}	-13.5 ± 4.2
Arg75^{HLA-E}	Asp163^{CD94}	-13.1 ± 2.4
Asp162^{HLA-E}	Arg213^{NKG2C}	-10.0 ± 5.6
Gln72^{HLA-E}	Glu164^{CD94}	-8.8 ±1.9
Asp162^{HLA-E}	Lys197^{NKG2C}	-8.4 ± 5.4
Glu166^{HLA-E}	Lys197^{NKG2C}	-7.4 ± 4.5
Asp162^{HLA-E}	Lys215^{NKG2C}	-5.6 ± 4.7
Arg108^{HLA-E}	Asp200^{NKG2C}	-5.1 ± 5.0
Glu154^{HLA-E}	Arg135^{NKG2C}	-3.8 ± 3.8
Glu152^{HLA-E}	Arg^{P5}	-3.8 ± 1.1
Arg75^{HLA-E}	Glu164^{CD94}	-3.5 ± 1.8
Peptide		
Arg^{P5}	Ser110^{CD94}	-5.7 ± 1.6
Thr^{P6}	Gln112^{CD94}	-3.9 ± 0.5

**COM$^{-}_{Cw7}$ ΔG_b -29.4 ± 12.5 kcal/mol**		
**ligand**	**receptor**	**ΔG_b total**
HLA-E		
Asp69^{HLA-E}	Arg171^{CD94}	-16.3 ± 1.3
Arg75^{HLA-E}	Asp163^{CD94}	-15.0 ± 2.4
Arg68^{HLA-E}	Glu164^{CD94}	-9.7 ± 4.8
Asp162^{HLA-E}	Lys197^{NKG2C}	-9.3 ± 4.8
Gln72^{HLA-E}	Glu164^{CD94}	-7.7 ± 1.4
Asp162^{HLA-E}	Lys215^{NKG2C}	-6.5 ± 3.8
Arg65^{HLA-E}	Gln79^{CD94}	-5.9 ± 1.9
Asp162^{HLA-E}	Arg213^{NKG2C}	-4.9 ± 4.2
Asn148^{HLA-E}	Arg135^{NKG2C}	-3.9 ± 1.2
Ser151^{HLA-E}	Arg135^{NKG2C}	-3.9 ± 1.1
Glu152^{HLA-E}	Arg^{P5}	-3.9 ± 0.9
Arg68^{HLA-E}	Asp168^{CD94}	-3.6 ± 3.6
Gln72^{HLA-E}	Phe114^{CD94}	-3.0 ± 0.4
Peptide		
Arg^{P5}	Ser110^{CD94}	-3.9 ± 1.8
Ala^{P6}	Gln112^{CD94}	-3.8 ± 1.2

Table S6. Molecular mechanics-generalized born surface area (MM-GBSA)[2] binding free energies (ΔG_b) between receptor (NKG2A/CD94) and ligand (HLA-E/β2m/peptide) and per-residue free energy decomposition (kcal/mol) calculated over time interval between 600 and 900 ns of the MD trajectory in the production run for **COM$^{+}_{G}$**, **COM$^{\sim}_{B27}$**, **COM$^{-}_{B7}$,** and **COM$^{-}_{Cw7}$** models. In the table are gathered first 10 ligand and receptor residues with the most favorable binding free energies.

**COM$^{+}_{G}$ ΔG_b -57.0 ± 15.3 kcal/mol**		**COM$^{\sim}_{B27}$ ΔG_b -52.3 ± 11.9 kcal/mol**		**COM$^{-}_{B7}$ ΔG_b -55.1 ± 13.1 kcal/mol**		**COM$^{-}_{Cw7}$ ΔG_b -29.4 ± 12.5 kcal/mol**	
**ligand**	**ΔG_b total**	**ligand**	**ΔG_b total**	**ligand**	**ΔG_b total**	**ligand**	**ΔG_b total**
HLA-E		*HLA-E*		*HLA-E*		*HLA-E*	
Asp69$^{\mathrm{HLA-E}}$	-7.4 ± 1.4	Asp162$^{\mathrm{HLA-E}}$	-9.2 ± 4.6	Glu166$^{\mathrm{HLA-E}}$	-6.4 ± 3.1	Asp69$^{\mathrm{HLA-E}}$	-6.6 ± 1.2
Asp162$^{\mathrm{HLA-E}}$	-6.8 ± 4.5	Asp69$^{\mathrm{HLA-E}}$	-5.2 ± 1.5	Arg68$^{\mathrm{HLA-E}}$	-5.9 ± 2.3	Asp162$^{\mathrm{HLA-E}}$	-4.8 ± 3.4
Arg68$^{\mathrm{HLA-E}}$	-5.2 ± 2.9	Gln72$^{\mathrm{HLA-E}}$	-3.7 ± 1.6	Asp69$^{\mathrm{HLA-E}}$	-5.5 ± 1.4	Gln72$^{\mathrm{HLA-E}}$	-3.6 ± 1.3
Glu154$^{\mathrm{HLA-E}}$	-4.7 ± 3.6	Arg79$^{\mathrm{HLA-E}}$	-3.7 ± 1.7	Asp162$^{\mathrm{HLA-E}}$	-5.3 ± 3.5	Arg75$^{\mathrm{HLA-E}}$	-3.6 ± 1.8
Gln72$^{\mathrm{HLA-E}}$	-3.5 ± 1.3	Arg68$^{\mathrm{HLA-E}}$	-3.0 ± 2.5	Gln72$^{\mathrm{HLA-E}}$	-4.2 ± 1.3	Arg68$^{\mathrm{HLA-E}}$	-2.4 ± 2.8
Glu166$^{\mathrm{HLA-E}}$	-2.6 ± 3.5	His155$^{\mathrm{HLA-E}}$	-2.5 ± 0.8	Arg79$^{\mathrm{HLA-E}}$	-2.7 ± 3.0	Ser151$^{\mathrm{HLA-E}}$	-2.3 ± 0.9
Arg75$^{\mathrm{HLA-E}}$	-2.5 ± 2.3	Arg65$^{\mathrm{HLA-E}}$	-2.4 ± 2.6	His155$^{\mathrm{HLA-E}}$	-2.6 ± 1.6	Arg62$^{\mathrm{HLA-E}}$	-2.2 ± 3.4
Ala158$^{\mathrm{HLA-E}}$	-1.8 ± 1.1	Arg75$^{\mathrm{HLA-E}}$	-2.0 ± 2.3	Arg108$^{\mathrm{HLA-E}}$	-2.4 ± 2.7	Ile73$^{\mathrm{HLA-E}}$	-2.2 ± 0.6
Peptide		Ile73$^{\mathrm{HLA-E}}$	-1.7 ± 0.4	Arg62$^{\mathrm{HLA-E}}$	-2.3 ± 2.9	Glu152$^{\mathrm{HLA-E}}$	-2.1 ± 1.3
Phe$^{\mathrm{P8}}$	-5.4 ± 1.1	*Peptide*		Glu152$^{\mathrm{HLA-E}}$	-2.3 ± 1.5	*Peptide*	
Thr$^{\mathrm{P6}}$	-2.2 ± 0.5	Leu$^{\mathrm{P8}}$	-2.6 ± 0.5	*Peptide*		Ala$^{\mathrm{P6}}$	-2.0 ± 1.1
**Receptor**	**ΔG_b total**	**Receptor**	**ΔG_b total**	**Receptor**	**ΔG_b total**	**Receptor**	**ΔG_b total**
CD94		*CD94*		*CD94*		*CD94*	
Asp163$^{\mathrm{CD94}}$	-11.4 ± 5.3	Asp163$^{\mathrm{CD94}}$	-10.0 ± 4.2	Glu164$^{\mathrm{CD94}}$	-9.6 ± 4.5	Arg171$^{\mathrm{CD94}}$	-4.9 ± 0.9
Glu164$^{\mathrm{CD94}}$	-7.1 ± 3.5	Arg171$^{\mathrm{CD94}}$	-4.9 ± 1.0	Arg171$^{\mathrm{CD94}}$	-5.1 ± 1.0	Phe114$^{\mathrm{CD94}}$	-4.7 ± 0.5
Phe114$^{\mathrm{CD94}}$	-5.1 ± 0.6	Phe114$^{\mathrm{CD94}}$	-4.6 ± 0.6	Phe114$^{\mathrm{CD94}}$	-4.8 ± 0.6	Asp163$^{\mathrm{CD94}}$	-4.5 ± 1.0
Arg171$^{\mathrm{CD94}}$	-4.0 ± 1.1	Glu164$^{\mathrm{CD94}}$	-4.3 ± 3.6	Asp163$^{\mathrm{CD94}}$	-3.9 ± 1.2	Glu164$^{\mathrm{CD94}}$	-4.4 ± 3.1
Asp168$^{\mathrm{CD94}}$	-3.1 ± 2.2	Ser110$^{\mathrm{CD94}}$	-3.0 ± 0.7	Ser110$^{\mathrm{CD94}}$	-3.2 ± 1.1	Ser110$^{\mathrm{CD94}}$	-1.9 ± 1.3
Thr146$^{\mathrm{CD94}}$	-1.9 ± 1.7	Thr146$^{\mathrm{CD94}}$	-1.6 ± 1.3	Leu162$^{\mathrm{CD94}}$	-1.8 ± 0.5	Gln79$^{\mathrm{CD94}}$	-1.7 ± 1.6
NKG2C		Leu162$^{\mathrm{CD94}}$	-1.5 ± 0.8	*NKG2C*		Leu162$^{\mathrm{CD94}}$	-1.5 ± 0.6
Arg213$^{\mathrm{NKG2C}}$	-5.9 ± 2.8	*NKG2C*		Arg213$^{\mathrm{NKG2C}}$	-8.8 ± 3.4	*NKG2C*	
Pro169$^{\mathrm{NKG2C}}$	-2.4 ± 0.7	Arg213$^{\mathrm{NKG2C}}$	-3.6 ± 1.7	Pro169$^{\mathrm{NKG2C}}$	-2.1 ± 0.6	Met223$^{\mathrm{NKG2C}}$	-2.4 ± 0.5
Arg135$^{\mathrm{NKG2C}}$	-2.1 ± 2.5	Pro169$^{\mathrm{NKG2C}}$	-2.4 ± 0.4	Lys197$^{\mathrm{NKG2C}}$	-1.1 ± 1.5	Pro169$^{\mathrm{NKG2C}}$	-2.1 ± 0.5
Ile224$^{\mathrm{NKG2C}}$	-1.5 ± 0.8	Ser170$^{\mathrm{NKG2C}}$	-1.3 ± 0.4	Arg135$^{\mathrm{NKG2C}}$	-1.1 ± 1.9	Ile224$^{\mathrm{NKG2C}}$	-1.5 ± 0.3

Table S7. Comparison of the binding free energies (ΔG_b) and interaction energies between ligand HLA-E/β2m/peptide and receptor NKG2C/CD94 calculated by a) Molecular mechanics-generalized born surface area (MM-GBSA)[2] (kcal/mol) and by b) gmx energy module of the Gromacs2016[3] software package (kJ/mol) approaches.

a)

Model	VDW [kcal/mol]	EEL [kcal/mol]	EGB [kcal/mol]	ESURF [kcal/mol]	ΔG_b [kcal/mol]
COM^{+}_{G}	-107.9 ± 1.0	-671.4 ± 8.0	741.1 ± 7.0	-18.8 ± 0.1	-57.0 ± 1.5
$COM^{\sim}_{B27}$	-92.1 ± 0.8	-614.6 ± 8.5	670.9 ± 7.6	-16.6 ± 0.1	-52.3 ± 1.2
COM^{-}_{B7}	-94.0 ± 0.9	-674.9 ± 7.6	731.4 ± 6.9	-17.6 ± 0.1	-55.1 ± 1.3
COM^{-}_{Cw7}	-99.7 ± 0.7	-585.0 ± 6.7	672.1 ± 6.4	-16.9 ± 0.1	-29.4 ± 1.2
COM_{apo}	-95.7 ± 7.9	-521.7 ± 84.0	595.9 ± 79.2	-16.0 ± 1.1	-37.6 ± 14.2

b)

Model	VDW [kJ/mol]	EEL [kJ/mol]	ΔG_b [kJ/mol]	ΔG_b [kcal/mol]
COM^{+}_{G}	-1142.4 ± 186.0	-305.8 ± 391.9	-1448.2 ± 577.9	-345.9 ± 138.0
$COM^{\sim}_{B27}$	-1137.9 ± 199.9	-270.0 ± 34.3	-1407.9 ± 234.2	-336.3 ± 55.9
COM^{-}_{B7}	-1201.0 ± 231.6	-265.4 ± 33.1	-1466.5 ± 264.7	-350.3 ± 63.2
COM^{-}_{Cw7}	-1073.6 ± 165.4	-283.5 ± 30.0	-1357.2 ± 195.4	-324.2 ± 46.7
COM_{apo}	-929.1 ± 170.4	-255.3 ± 32.0	-1184.4 ± 202.4	-282.9 ± 48.3

Supplementary Movie

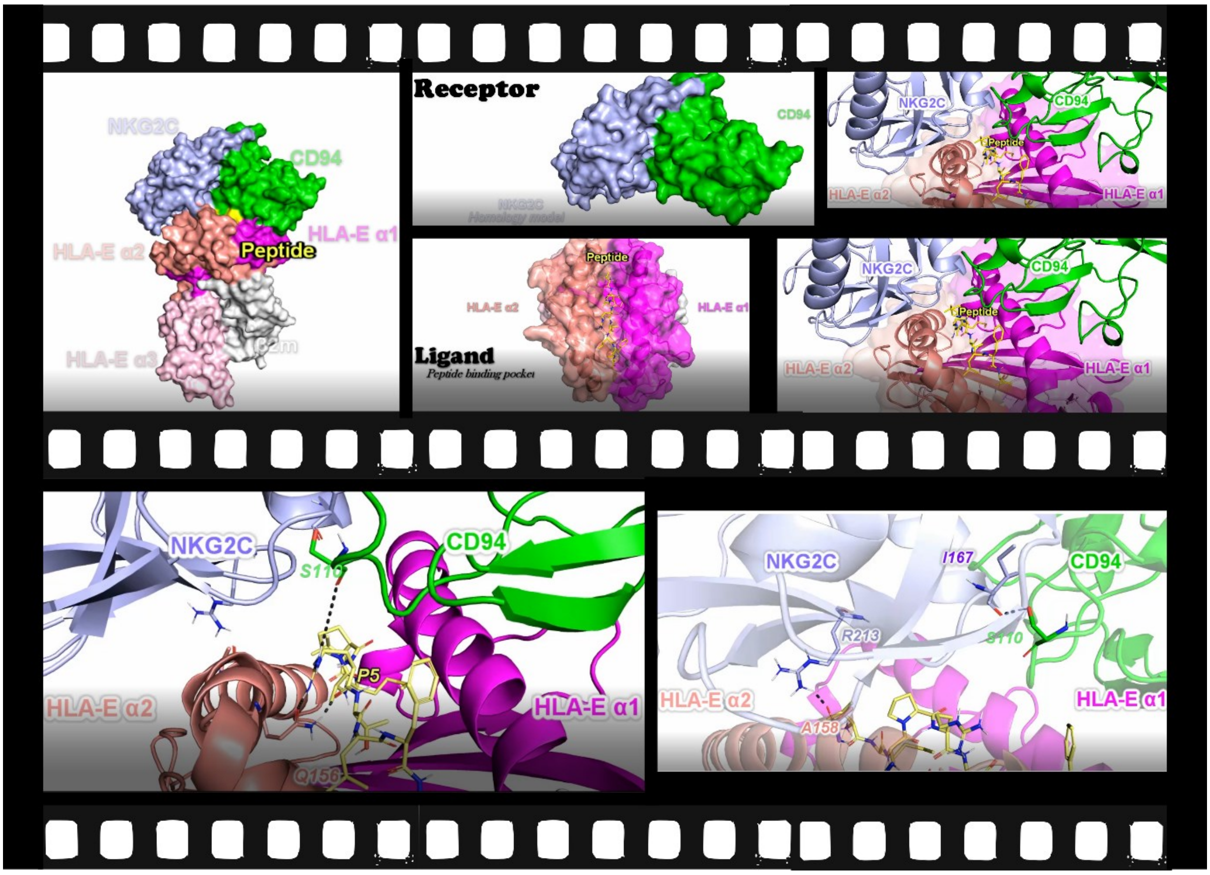

Movie S1. The presentation of the immune complex HLA-E/β2m/peptide/NKG2C/CD94 including the receptor and ligand part, placement of the nonameric peptide between HLA-E α1 and 2 domains, and identified key interactions.

Supplementary References

[1] E. J. Petrie, C. S. Clements, J. Lin, L. C. Sullivan, D. Johnson, T. Huyton, A. Heroux, H. L. Hoare, T. Beddoe, H. H. Reid, et al., *J. Exp. Med.* **2008**, *205*, 725–735.

[2] I. Massova, P. A. Kollman, *Perspect Drug Discov Des* **2000**, *18*, 113–135.

[3] D. Van Der Spoel, E. Lindahl, B. Hess, G. Groenhof, A. E. Mark, H. J. C. Berendsen, *J Comput Chem* **2005**, *26*, 1701–1718.
